# Genetic fusions favor tumorigenesis through degron loss in oncogenes

**DOI:** 10.1038/s41467-021-26871-y

**Published:** 2021-11-18

**Authors:** Jing Liu, Collin Tokheim, Jonathan D. Lee, Wenjian Gan, Brian J. North, X. Shirley Liu, Pier Paolo Pandolfi, Wenyi Wei

**Affiliations:** 1 Department of Pathology, Beth Israel Deaconess Medical Center, Harvard Medical School, Boston, MA 02215 USA; 2 Department of Data Science, Dana-Farber Cancer Institute, Boston, MA 02215 USA; 3 Department of Biostatistics, Harvard T.H. Chan School of Public Health, Boston, MA 02115 USA; 4 Cancer Research Institute, Beth Israel Deaconess Cancer Center, Department of Medicine and Pathology, Beth Israel Deaconess Medical Center, Harvard Medical School, Boston, MA 02215 USA; 5 Paulson School of Engineering and Applied Sciences, Harvard University, Cambridge, MA 02138 USA; 6 Department of Biochemistry and Molecular Biology, Medical University of South Carolina, Charleston, SC 29425 USA; 7 Department of Biomedical Sciences, Creighton University, Omaha, NE 68178 USA; 8 Department of Molecular Biotechnology and Health Sciences, University of Turin, Turin, 10124 Italy; 9 Renown Institute for Cancer, Nevada System of Higher Education, Reno, NV 89502 USA

**Keywords:** Cancer genomics, Ubiquitin ligases, Ubiquitylation, Cancer genetics, Oncogenes

## Abstract

Chromosomal rearrangements can generate genetic fusions composed of two distinct gene sequences, many of which have been implicated in tumorigenesis and progression. Our study proposes a model whereby oncogenic gene fusions frequently alter the protein stability of the resulting fusion products, via exchanging protein degradation signal (degron) between gene sequences. Computational analyses of The Cancer Genome Atlas (TCGA) identify 2,406 cases of degron exchange events and reveal an enrichment of oncogene stabilization due to loss of degrons from fusion. Furthermore, we identify and experimentally validate that some recurrent fusions, such as BCR-ABL, CCDC6-RET and PML-RARA fusions, perturb protein stability by exchanging internal degrons. Likewise, we also validate that EGFR or RAF1 fusions can be stabilized by losing a computationally-predicted C-terminal degron. Thus, complementary to enhanced oncogene transcription via promoter swapping, our model of degron loss illustrates another general mechanism for recurrent fusion proteins in driving tumorigenesis.

## Abstract (web summary)

The impact of genetic fusions on degrons, which are motifs for ubiquitin-mediated protein degradation, has not been fully explored. Here, the authors analyse fusion genes affecting degrons in pan-cancer genomics data, validate their functional impact and find enrichment for both internal and C-terminal degron losses.

## Introduction

Genetic alterations accumulate during the multistep processes of tumorigenesis, which lead to the transformation of normal cells into cancer cells^1,2^. Large-scale tumor sequencing has enabled the systematic identification of gene fusions derived from chromosomal rearrangements. The most famous chromosomal rearrangement, t(9;22), was identified in 1960 as a hallmark of chronic myeloid leukemia (LCML) and subsequently named the Philadelphia chromosome^3^. The Philadelphia chromosome promptes the discovery of the BCR (breakpoint cluster region)-ABL fusion^4,5^ and the clinical application of imatinib as a targeted therapy for treating LCML patients^6^. To date, chromosomal rearrangements have been reported as frequent genetic drivers of several types of human cancer, such as ETS-related gene (ERG) fusions in prostate cancer^7^, RET or anaplastic lymphoma kinase (ALK) fusions in lung cancer^8,9^, and fibroblast growth factor receptor 3 (FGFR3) fusions in bladder cancer^10^. According to a previous comprehensive analysis of The Cancer Genome Atlas (TCGA), there are more than 25,000 genetic fusion events, which might drive the development of approximately 16.5% of total cancer cases^11^. However, the molecular mechanisms underlying how these gene fusions are oncogenic remain largely unclear for most of these cases.

Several mechanisms have been proposed to explain the oncogenicity of fusion proteins^12^. One mechanism relies on transcriptional up-regulation due to promoter exchange between two genes, such as the fusion of ERG with the 5′-UTR of *TMPRSS2 (transmembrane serine protease 2)* to trigger the transcription of fusion products in prostate cancer^13^. Another mechanism for the oncogenic nature of fusion proteins is the constitutive activation of kinases, often achieved by dimerization or oligomerization, such as for ABL, ALK, and RET fusions^5,8,9,14^. A third mechanism is the loss of an auto-inhibitory segment, such as for BRAF fusions^15^. We hypothesized that altered protein stability could be an additional widespread mechanism for gene fusion events. While there have been a few instances characterized, such as TMPRSS2-ETV1^16^ and TMPRSS2-ERG fusions^17,18^, altered protein stability has not been previously discussed as a major mechanism for the functional impact of gene fusions on tumorigenesis^12^.

 Intracellular protein homeostasis is strictly controlled by the balance between protein synthesis by the ribosome and protein degradation by the ubiquitin proteasome system (UPS)^19^. Proteins are targeted for 26S proteasome-mediated proteolysis by conjugation of a poly-ubiquitin chain onto lysine residues^20,21^. The exquisite selectivity of the ubiquitination process on a cellular protein relies on its recognition by specific E3 ubiquitin ligase(s)^22–24^. There are more than 600 E3 ligases encoded in the human genome, but only a few have been extensively characterized^22–24^. The binding specificity of an E3 ligase is thought to be governed by short sequence motifs on the substrate, known as degrons^25,26^, which are typically several amino acids long. Some E3 ligases display strong locational preference for degrons at the C-terminus or N-terminus of a protein^27–29^, while other degrons can be found within the internal protein sequence^26^.

While our recent analysis suggest that oncogenic point mutations frequently perturb the function of the UPS (~19% of cancer driver genes)^30^, it remains unknown whether gene fusions also frequently alter protein stability. In this study, we identify 2406 fusion candidates with possible degron loss preferentially occurred in oncogenes (OG) from bioinformatics analysis across 33 cancer types, and further experimentally validate the increased protein stability resulting from loss of degrons in 5 fusions, indicating that altering protein stability due to degron loss is a general mechanism for cancer-related genetic fusions to promote tumorigenesis.

## Results

### A systematic computational analysis of degron loss

**Fig. 1:**
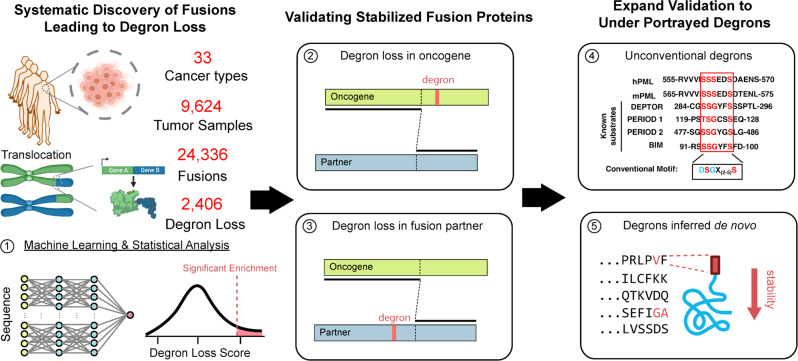
Degron loss-prone genetic fusion events favor oncoprotein stabilization and tumorigenesis. A schematic diagram to illustrate the mechanism of how gene fusion leads to degron gain/loss event and thus perturbs proteins stability. Created with BioRender.com.

 Previous reports of degron loss in gene fusions have largely focused on prostate cancer due to the high frequency of oncogenic fusions^7,11,16^. The two most common fusion events in prostate cancers involve either ERG (>50% of primary tumor samples) or ETV family transcription factors (<10%). Notably, we and others have reported that through the fusions with TMPRSS2 or other 5′ partners, ERG loses an SPOP (speckle-type POZ protein) degron, thus leading to stabilization of the fusion protein (Supplementary Fig. 1a, b)^17,18^. Similarly, ETVs also lose two COP1 degrons during fusion, which leads to escape from COP1-mediated degradation (Supplementary Fig. 1c–f)^16^. These studies prompted us to hypothesize that degron loss could be a general mechanism for genetic fusion events in driving tumorigenesis beyond prostate cancer (Fig. 1).

**Fig. 2:**
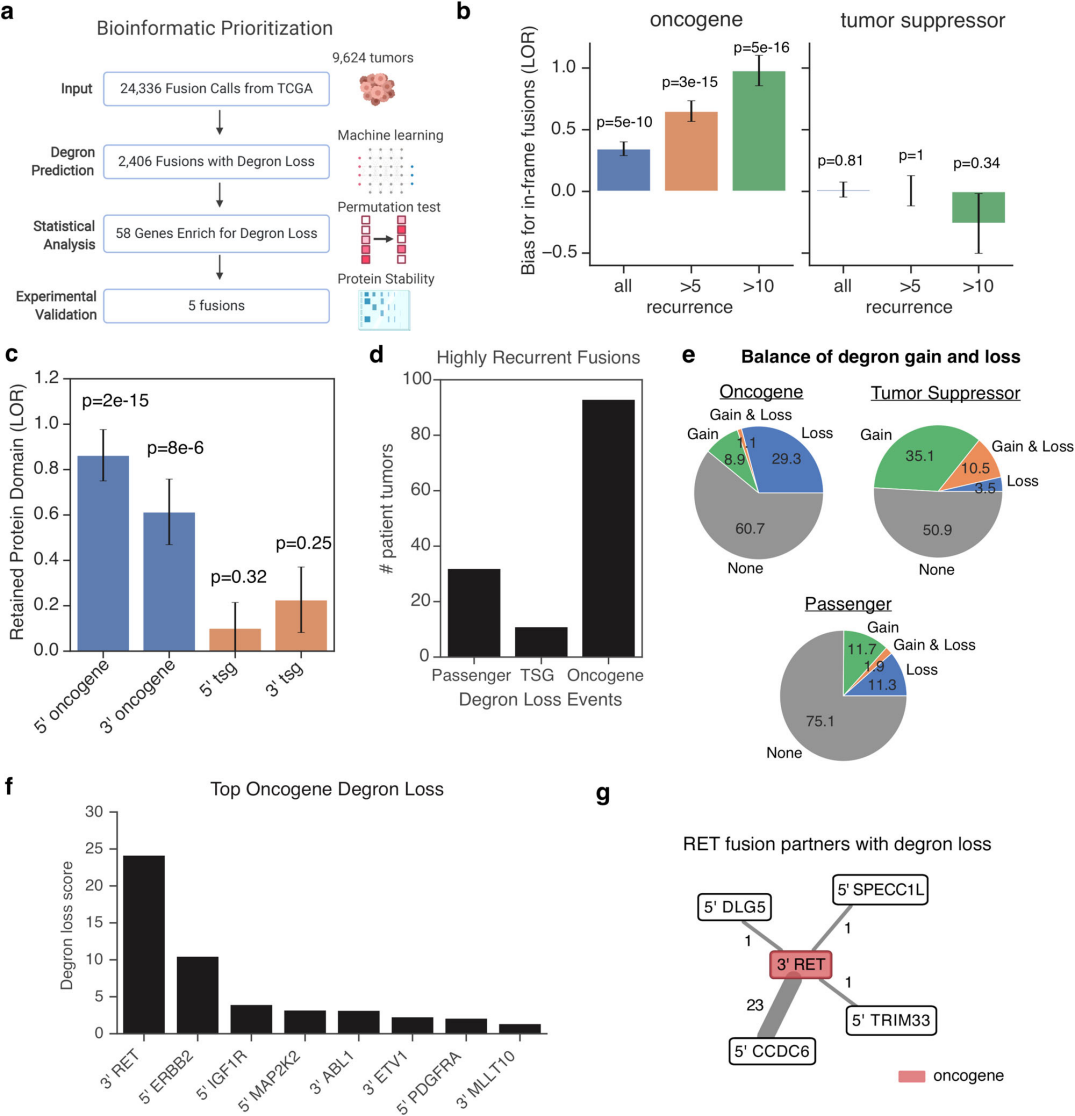
Degron landscape of genetic fusions in cancer. **a** Combined analysis pipeline for validating cases of degron loss in fusion genes. Created with BioRender.com. **b** Enrichment for in-frame gene fusions for oncogenes and tumor suppressor genes relative to likely passenger genes (STAR methods). Recurrence is the number of tumors that a fusion gene was present in the TCGA. Barplot displays the log odds ratio (LOR), with error bars indicating ±1 SE. In order of bars from left to right, sample sizes (i.e. number of fusions) are *n* = 17,848, *n* = 4254, *n* = 1316, *n* = 17,581, *n* = 3,799, and *n* = 925. A two-sided Fisher’s exact test was performed. **c** Enrichment analysis for whether gene fusions result in the retention of at least one protein domain from the original protein sequence. Barplot displays the log odds ratio (LOR), with error bars indicating ±1 SE. In order of bars from left to right, sample sizes (i.e. number of fusions) are *n* = 8911, *n* = 9139, *n* = 8935, and *n* = 8896. A two-sided Fisher’s exact test was performed. **d** The total number of tumors in the TCGA that contain highly recurrent fusion genes (a fusion found in >10 tumors). **e** Balance of degron gain and loss in oncogene, tumor suppressor genes or passenger genes. **f** The top 10 most significant oncogenes that are biased towards loss of internal degrons in gene fusions. **g** Network diagram showing degron loss in the fusion partners to oncogene RET. Line width represents the frequency of the event. Red indicates previously annotated oncogenes. Numbers indicate the recurrence of the fusion across TCGA. The relevant raw data are provided in Source Data.

We therefore systematically analyzed 24,336 fusion genes reported across 9624 tumor samples in TCGA to discern the importance of a degron loss mechanism (Fig. 2a and Supplementary Data 1). Consistent with a likely substantial contribution of gain-of-function fusions towards tumorigenesis in TCGA, we found that fusions containing previously implicated oncogenes were enriched for in-frame fusions (*p* < 5 × 10^−10^, Fig. 2b) and preferentially retained functional protein domains (*p* < 8 × 10^−6^, Fig. 2c). To understand the specific contribution of degron loss, we systematically predicted internal degrons for E3 ligases with known motifs using a Random Forest machine learning model (Supplementary Fig. 2a–d, “Methods”). In addition, we also unbiasedly predicted C-terminal degrons using the deepdegron method that we previously developed^30^ to identify degron motifs from the global protein stability assay^31^. Notably, among the highly recurrent fusions (>10 tumor samples), degron loss is significantly more enriched in oncogenes (Fig. 2d, e, 30.4%) than tumor suppressor genes (Fig. 2d, e, 14%, *p* = 0.01, Fisher’s exact test) or likely passenger genes (Fig. 2d, e, 13.2%, *p* = 5 × 10^−6^). These results were robust to the choice of threshold for recurrent fusions (Supplementary Fig. 2e). Moreover, fusions involving oncogenes displayed a clear bias for degron loss over degron gain (Fig. 2e). In contrast to oncogenes, fusions involving tumor suppressor genes had a trend towards degron gain, although the overall number of events was relatively low. Taken together, these results indicate that degron loss could be a major contributor to the oncogenicity of gene fusions.

We next systematically discovered the specific genes involved in fusions that preferentially underwent degron loss. By first analyzing internal degrons, we identified 47 genes where gene fusions led to more predicted loss of internal degrons than expected (*q* < 0.1, permutation test, “Methods”^32^), which contained several known oncogenes, such as ABL1, RET, and IGF1R (insulin like growth factor 1 receptor) fusions (Fig. 2e, Supplementary Fig. 2f, g and Supplementary Data 2). Likewise, genes that are fusion partners to well-known oncogenes were also common (Fig. 2g and Supplementary Fig. 2h), such as the statistically significant degron loss for CCDC6, particularly when fused with RET (*p* < 0.0001, permutation test). This suggested a potential selection pressure to avoid protein degradation in both members of a fusion gene product. By further restricting our analysis to only previously implicated oncogenes (*q* < 0.1), we found additional internal degron loss events for rare oncogenic fusions containing PDGFRA/FIP1L1 (platelet-derived growth factor receptor alpha/factor interacting with PAPOLA and CPSF1, Supplementary Fig. 2i) and a positive control ETV1 fusion (Supplementary Fig. 1c and Supplementary Data 2), with additional fusions containing ETV4 (*q* = 0.16, Supplementary Fig. 1e) and ETV5 (*q* = 0.13, Supplementary Fig. 1f) at the borderline of statistical significance. Thus, it is plausible that with greater sample size, additional fusions leading to degron loss in genes not previously known to be oncogenes will be found.

### Genetic fusions with degron loss are likely cancer type-specific

**Fig. 3:**
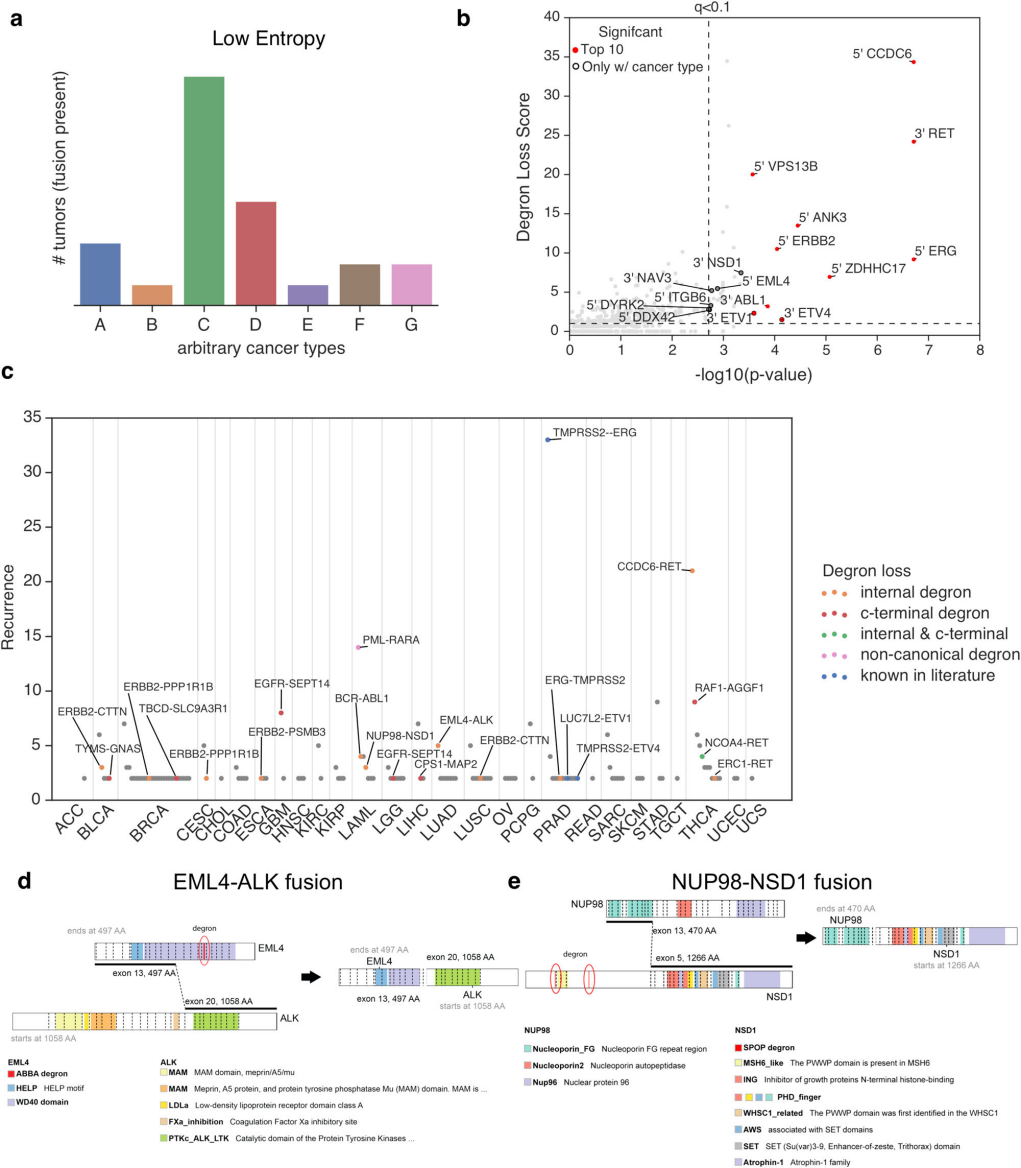
Cancer type-specificity of genetic fusions reveals oncogenicity. **a** Conceptual diagram showing that a low entropy value that summarizes the distribution of a fusion across cancer types indicates the fusion gene is cancer type-specific. **b** Scatter plot showing the statistical significance of gene fusions for loss of internal degrons when also considering information on cancer type-specificity. *X*-axis represents the combined *p* value for both degron loss and cancer type-specificity (Fisher’s method), while the *Y*-axis represents the effect size for degron loss. *P* values were calculated using a one-sided permutation test. See “Methods” for additional details on calculation of *p* values. **c** Scatter plot indicating the most recurrent fusion genes for particular cancer types. Fusion genes with degron loss are labeled in color, including internal degrons, C-terminal degrons, and those degrons previously known in the literature. **d** A schematic diagram showing the fusion of *EML4* and *ALK* genes in lung adenocarcinoma. **e** A schematic diagram showing the fusion of *NUP98* and *NSD1* genes in lung adenocarcinoma. The relevant raw data are provided in Source Data.

Given that genetic fusions have been previously noted to exhibit tissue specificity, such as ETV family fusions in prostate cancer^7^ and ALK or MET fusions in lung cancer^8,9,14^, we hypothesized that inclusion of cancer type-specificity would likely improve our statistical power. To this end, using low entropy as a metric for specificity (Fig. 3a), we found that genes involved in highly cancer type-specific fusions were significantly enriched for previously known oncogenic fusions (*p* = 3.1 × 10^−16^, Fischer’s Exact test, Supplementary Fig. 3a and Supplementary Data 3). Interestingly, we observed statistical significance for the loss of internal degrons from several fusion genes, such as 5′ EML4 fusions, 3′ NSD1 fusions and the previously validated 3′ ETV4 fusions, only when considered in conjunction with cancer type (Fig. 3b and Supplementary Data 3). Overall, degron loss contributes to many of the most highly recurrent gene fusions specific to particular cancer types (Fig. 3c), including PML-RARA in acute myeloid leukemia (LAML), EGFR-SEPT14 in gliomas, and TMPRSS2-ERG in prostate cancers. Thus, by using an unbiased statistical approach, we found both known (e.g. ETV fusions) and previously unknown cases of gene fusions leading to degron loss.

As numerous gene fusions with degron loss exhibit cancer type-specificity, we sought to identify the corresponding E3 ligases likely involved in this specificity. These associations include APC/CDC20 for EML4-ALK fusions in lung cancers (Fig. 3d), SPOP for NUP98-NSD1 (Fig. 3e) and BCR-ABL fusions in LAML, and FBW7 (or FBXW7, F-box and WD repeat domain containing 7) for CCDC6-RET fusions in thyroid carcinomas (THCAs) (Supplementary Data 3). Indeed, the E3 ligase most frequently involved in degron loss is the known tumor suppressor gene SPOP (Supplementary Fig. 3b), which suggests a selective pressure to avoid protein degradation in a variety of cancer types.

### Genetic fusions with degron loss are associated with downstream functional consequences

Based on the above analyses that degron loss may lead to increases in the stability of fusion proteins, we hypothesized that tumors containing these fusions would be associated with an altered proteomic and subsequent transcriptomic state of cancer cells. To validate this hypothesis, we first analyzed the abundance of 198 proteins measured by reverse phase protein arrays (RPPA) across the TCGA (Supplementary Data 4). Consistent with our finding of degron loss, 5′ ERBB2 and 5′ EGFR fusions had significantly higher expression levels and active phosphorylation of their respective proteins (Supplementary Fig. 3d). Furthermore, degron loss in CCDC6 fusions led to elevated levels of downstream effectors, including active phosphorylated forms of YAP, PKC, p38, and 4EBP1 (Supplementary Fig. 3d). Given the limited number of proteins assayed by RPPA, we next analyzed for potential downstream consequences on the transcriptome through modulating the activity of transcription factors (TFs). Since many oncogenic fusions are involved in protein signaling, we reasoned that TF activity could be best approximated by the expression of TF target genes. Here, TF target genes are defined by thousands of ChIP-seq profiles from the Cistrome database^33^. Using the RABIT algorithm^34^ to find coordinated differential expression of TF target genes, we found 113 significant associations between TF activity and fusion events (Supplementary Fig. 3e, f and Supplementary Data 5). In support of the reliability of our analysis, previous studies support several of the most significant associations identified, such as AR for ERG fusions, TTF1 for EML4-ALK fusions, and TAL1 for BCR-ABL fusions^35–37^. Interestingly, 5′ EGFR fusions were significantly associated with increased STAT1 activity, suggesting that it is either a downstream consequence of EGFR kinase activity or an immunogenic consequence of a predicted fusion neoantigen^11,38^. Cumulatively, our analyses indicate that fusion events undergoing degron loss have significant downstream functional consequences on both the proteome as well as the transcriptome.

### BCR-ABL fusion leads to loss of the SPOP degron in ABL and stabilization of fusion protein

**Fig. 4:**
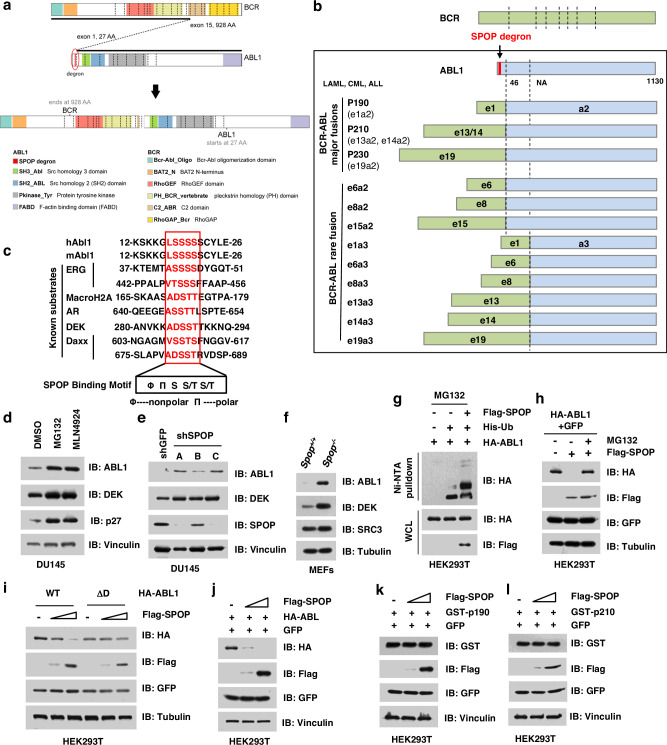
BCR-ABL escapes from SPOP-mediated degradation due to degron loss. **a** A schematic diagram showing the fusion of *BCR* and *ABL* genes in chronic myeloid leukemia (LCML). **b** BCR-ABL fusion leads to the loss of a SPOP degron (-LSSSS-) on the N-terminus of ABL protein, resulting in stabilization of BCR-ABL fusion proteins, including p190, p210, p230, and other rare fusions. **c** SPOP degron in ABL1 is conserved among species. Φ: nonpolar; Π: polar. **d** The degradation of ABL1 is proteasome- and Cullin E3 ligase-dependent. DU145 cells were treated with either 10 μM MG132 or 1 μM MLN4924 for 12 h. Cell were harvested, lysed, and immunoblotted for ABL1 with ERG and p27 as positive controls. **e** Depletion of *SPOP* leads to accumulation of ABL1 in DU145 cells. **f** *Spop*^*−/−*^ MEFs has relatively higher protein abundance of ABL1 than WT MEFs. **g** SPOP promotes the ubiquitination of ABL1 in cells. HEK293T cells were transfected with indicated constructs and treated with 30 μM MG132 for 6 h. Cells were lysed under denaturing conditions and His-Ub-conjugated proteins were pulled down with Ni-NTA-resin, washed and IB for indicated proteins. **h** SPOP promotes the degradation of ABL1 in a proteasome-dependent manner. HEK293 cells were transfected with indicated constructs and treated with or without 10 μM MG132 for 12 h, and then harvested, lysed, and immunoblotted for indicated proteins. **i** Deletion of the SPOP-binding motif in ABL1 leads to stabilization of ABL1 protein. Cells were transfected with indicated constructs for 48 h, followed by harvested, lysed, and immunoblotted for indicated proteins. **j**–**l** Compared with ABL-WT, BCR-ABL fusion proteins, both p190 and p210, which lack the SPOP degron, were relatively resistant to SPOP-mediated degradation. Cells were transfected increasing concentrations of Flag-SPOP together with either ABL-WT (**j**), BCR-ABL fusion proteins, both p190 (**k**) and p210 (**l**). Cells were then harvested, lysed, and IB of indicated proteins. Two independent experiments were performed for **d**–**l**. The relevant raw data and uncropped blots are provided in Source Data.

Our systematic computational approach allowed us to potentially find, even for the most well-studied oncogenes, previously unknown degrons that were lost during fusion. For example, our analyses predicted that BCR-ABL fusions led to the loss of a SPOP degron originally found in the oncoprotein ABL1 (Fig. 4a). BCR-ABL is the gene fusion product of the Philadelphia chromosome found in LCML (Supplementary Fig. 4a, b)^3–5^, and it has been a therapeutic target for LCML treatment for decades^6,39,40^. Our computational analysis predicted that the fusion between ABL and its 5′ partner BCR leads to loss of a degron recognized by SPOP (Fig. 4a, b), which is a substrate adaptor of the Cullin 3 family of E3 ligases. The putative SPOP degron (17-LSSSS-21) is evolutionarily conserved in human and mouse ABL1 protein sequence, and similar to several known SPOP substrates, including ERG, AR, and DEK (Fig. 4c)^17,18,41^. This indicated that SPOP degron loss was plausible for BCR-ABL fusions, and thus might complement a previously proposed mechanism of constitutive kinase activity^42^.

First, we aimed to experimentally validate ABL1 as a bona fide substrate of the Cullin 3^SPOP^ E3 ligase. Indeed, similar to the known SPOP substrate ERG, the protein abundance of ABL1 increased in DU145 prostate cancer cells upon treatment with either the proteasome inhibitor MG132 or the neddylation inhibitor MLN4924 (Fig. 4d). Depletion of endogenous *Cullin 3* (Supplementary Fig. 4c) or *SPOP* (Fig. 4e) led to an increase of ABL1 protein abundance. Furthermore, *Spop*^*−/−*^ mouse embryonic fibroblasts (MEFs) had relatively higher protein abundance of ABL1 than wild-type (WT) MEFs (Fig. 4f), consistent with the positive control SPOP substrates DEK^41^ and SRC3 (ref. ^43^). As expected for abrogating protein degradation, the protein half-life of ABL1 was dramatically longer in *Spop*^*−/−*^ MEFs than in WT MEFs (Supplementary Fig. 4d, e). Moreover, ectopic expression of SPOP promoted the ubiquitination and degradation of ABL1 protein, which could be largely inhibited by the proteasome inhibitor MG132, thus indicating a proteasome-dependent mechanism (Fig. 4g, h). To ensure the enhanced degradation of the ABL1 protein was due to an on-target mechanism, we evaluated whether cancer-derived mutations, including Y87C, F102C, W131G, and F133V (Supplementary Fig. 4f)^44^, that abrogate SPOP binding to substrates would fail to promote ABL1 protein degradation. Notably, ectopic expression of WT SPOP, but not the SPOP mutants, could degrade ABL1 protein (Supplementary Fig. 4g). Taken together, these results support our computational prediction and indicate that ABL1 is likely a bona fide substrate of the SPOP E3 ligase.

We next sought to investigate whether BCR-ABL fusion proteins, named p190 and p210, could escape SPOP-mediated degradation in cells. This requires firstly excluding the possibility of another SPOP degron in ABL1 which is not lost in a fusion. We found that after deleting the predicted SPOP degron in the ABL1 protein, the resultant ABL1-ΔD mutant was relatively resistant to SPOP-mediated degradation in cells (Fig. 4i). Secondly, ectopic expression of SPOP degrades only WT ABL1 (Fig. 4j), but not BCR-ABL1 fusion proteins (Fig. 4k, l), indicating that BCR-ABL fusions escape from SPOP-mediated degradation via loss of the sole SPOP degron in the N-terminus of ABL1. Apart from the most frequent fusions, p190 and p210, there are several other low frequent fusions (e19a2) and rare fusions (e6a2, e8a2, e15a2, e1a3, e6a3, e8a3, e13a3, e14a3, and e19a3) in LCML, LAML, and acute lymphocytic leukemia (ALL, Fig. 4b). Notably, the SPOP degron in exon 1 of *ABL* is lost in all of these genetic fusions (Fig. 4b), suggesting a similar mechanism for these fusions in promoting tumorigenesis.

### CCDC6-RET fusion escapes from FBW7-mediated degradation

**Fig. 5:**
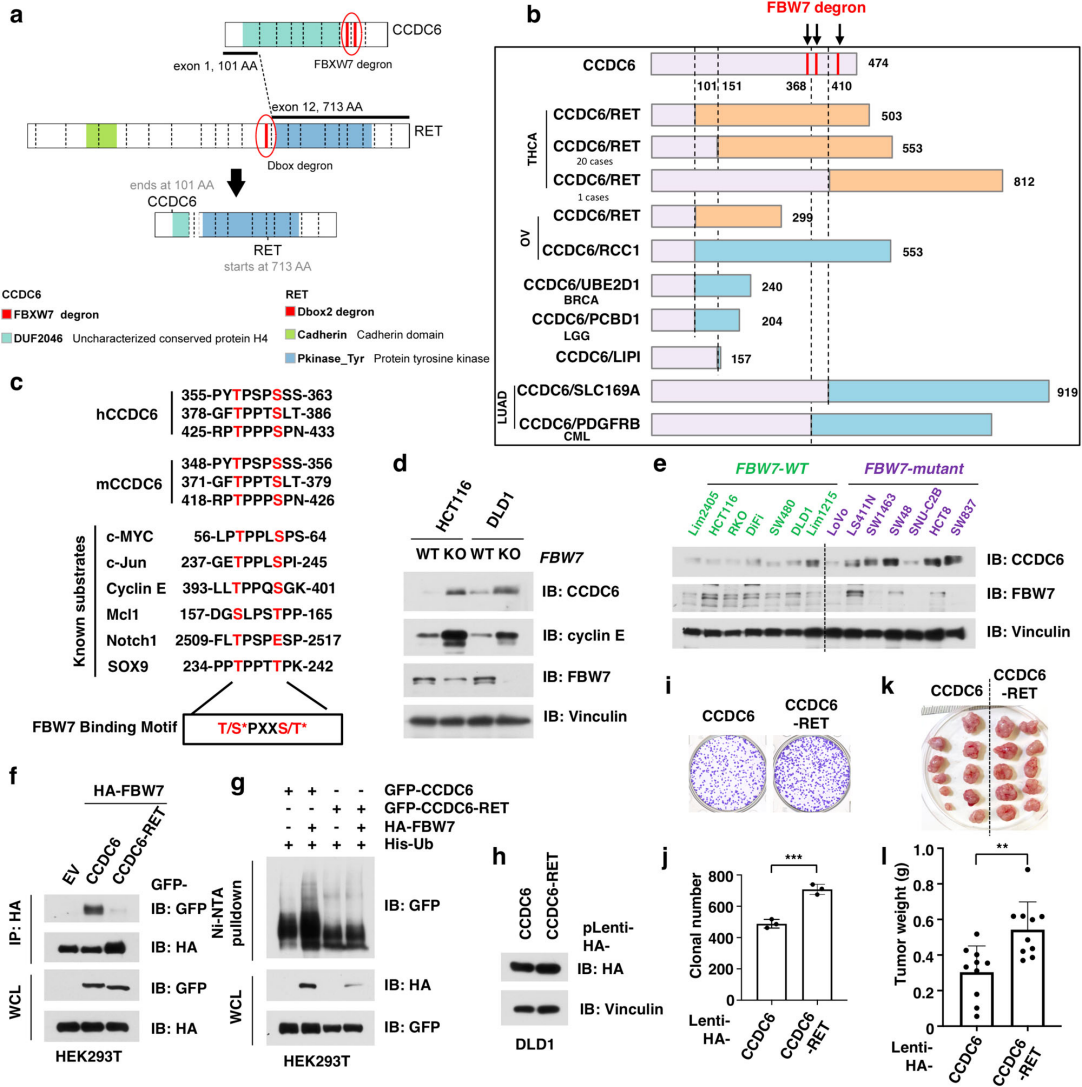
CCDC6-RET escapes from FBW7-mediated degradation due to degron loss during genetic fusion. **a** A schematic diagram shows the fusion of *CCDC6* and *RET* genes in thyroid carcinoma (THCA). **b** CCDC6-RET fusion events remove the FBW7 degron from the CCDC6 protein, leading to the stabilization of CCDC6-RET fusion proteins. **c** FBW7 degron of CCDC6 is evolutionarily conserved among species. *Phosphorylation. **d** Knockout of *FBW7* in DLD1 and HCT116 cells leads to accumulation of CCDC6 protein. DLD1 or HCT116 WT and *FBW7* deleted cells were harvested, lysed, and immunoblotted for indicated proteins. **e** Colorectal cancer (CRC) cells with *FBW7*-mutant exhibited relatively higher protein abundance of CCDC6, compared with those cells with WT *FBW7*. **f** CCDC6-RET fusion protein escaped from the recognition by the FBW7 E3 ligase. Cells were transfected with HA-FBW7 together with either GFP-tagged CCDC6 and CCDC6-RET fusion constructs. Cells were  treated with 10 μM MG132 for 12 h, followed by harvested, lysed, and immunoprecipitated (IP) with anti-HA. Inputs and immunoprecipitated material were immunoblotted for GFP and HA. **g** CCDC6-RET fusion protein escaped from FBW7-mediated ubiquitination. Cells were transfected with His-Ub, HA-FBW7 together with either GFP-tagged CCDC6 and CCDC6-RET fusion constructs. Cells were treated with 30 μM MG132 for 6 h, followed by lysed under denaturing conditions. The His-Ub-conjugated proteins were pulled down with Ni-NTA-resin, washed, and immunoblotted for indicated proteins. **h** Immunoblot analysis for DLD1 stable cell lines that ectopically express either wild-type (WT) CCDC6 or CCDC6-RET fusion protein. **i**, **j** CCDC6-RET-expressing cells had greater clonogenicity than WT-CCDC6-expressing cells in vitro. The DLD1 stable cell lines as in **h** were subjected for colony formation assay (**i**) and statistical analysis (**j**). *n* = 3/group. **k**, **l** CCDC6-RET-expressing cells developed larger tumors than WT-CCDC6-expressing cells in vivo. The DLD1 stable cell lines as in **h** were subjected for mouse xenograft assay (**k**) and statistical analysis (**l**). *n* = 10 mice/group. Data are presented as mean ± SD. **p < 0.01; ***p < 0.001; by two-tailed Student’s *t*-test. Two independent experiments were performed for **d** and **h**. The relevant raw data and uncropped blots are provided in Source Data.

Although the BCR-ABL fusion led to loss of a degron in the known oncoprotein ABL1, degron loss in the fusion partner to known oncogenes might also contribute towards increasing protein stability of fusion proteins. Our computational analysis showed that CCDC6-RET fusions were highly enriched for loss of predicted degrons in both fusion components, including FBW7 degrons in CCDC6 and a D-box degron in the oncogene RET (Fig. 5a). There are several variants of CCDC6-RET fusion, which contain N-terminal fragments of CCDC6 and C-terminus of RET, in thyroid carcinoma^45^, non-small cell lung cancer^9^, and other cancer types^46^ (Fig. 5b). Apart from *RET*, *CCDC6* also fuses with other genes, including *ROS1* (ref. ^47^) and *PDGFRB*^48^. Notably, the FBW7 degrons in CCDC6 were lost in all of these fusion proteins (Fig. 5b), suggesting an analogous mechanism of increasing protein stability.

Given these computational predictions, we expected the putative FBW7 degrons in CCDC6 would be similar to those found in previously known substrates. Sequence alignment showed that the predicted FBW7 degrons ((pT/pS)PXX(pS/pT), p indicating phosphorylation) were conserved in both human and mouse CCDC6, consistent with several known FBW7 substrates, such as c-Myc^49,50^, c-Jun^51^ and cyclin E^52^ (Fig. 5c). The recognition by FBW7 is known to dependent on the phosphorylation of serine or threonine residues within its degron motif^53^. As expected, large-scale phospho-proteomics data (https://www.phosphosite.org)^54^ have detected phosphorylation on residues within the putative FBW7 degrons (Thr-357, Ser-361, Thr-380, Ser-384, and Thr-427), further supporting CCDC6 as a potential substrate of FBW7.

To experimentally assess whether CCDC6-RET escapes FBW7-mediated degradation, we aimed to first validate CCDC6 as a bona fide FBW7 substrate. We found that the CCDC6 protein levels were relatively higher in *FBXW7 (*also known as *FBW7)* null DLD1 and HCT116 cells, compared with respective WT parental control cells (Fig. 5d). *FBW7* is frequently mutated and inactivated in colorectal cancer (CRC), and *FBW7* mutant CRC cells have relatively lower FBW7 expression and higher abundance of FBW7 substrates such as MCL1 (ref. ^55^). Thus, we further assessed CCDC6 protein levels in a panel of CRC cells with either WT or mutant *FBW7*, and found that *FBW7*-mutant cells trend to have relatively higher abundance of CCDC6 protein than *FBW7*-WT cells (Fig. 5e and Supplementary Fig. 4h). These data together indicate that CCDC6 is a ubiquitin substrate of FBW7. More importantly, compared with WT-CCDC6, CCDC6-RET fusion protein escaped recognition by FBW7 (Fig. 5f), leading to stabilization of the resultant fusion product in the in vivo ubiquitination assay (Fig. 5g). In keeping with this notion, depletion of *FBW7* extended the half-life of CCDC6 protein in a cycloheximide (CHX) chasing assay (Supplementary Fig. 4i, j).

Unlike a prior report of CCDC6 as a substrate of FBW7^56^, our findings support the relevance of FBW7 degron loss in CCDC6 fusions. Interestingly, given that CCDC6-RET fusions are predicted to generate neoantigens (Supplementary Fig. 4k)^11^, an increase of CCDC6-RET protein stability might also reduce the generation of antigenic peptides derived from proteasomal degradation^57^, thus evading an otherwise strong immune response (*p* = 0.02, likelihood ratio test; Supplementary Fig. 4l). To assess how loss of FBW7 degrons in the CCDC6 protein impact tumorigenesis, we further generated a DLD1 cell line that stably expresses either WT CCDC6 or CCDC6-RET fusion protein (Fig. 5h). We found that the CCDC6-RET-expressing cell line were more clonogenic than the WT-CCDC6-expressing cells in a colony formation assay (Fig. 5i, j) and resulted in larger tumors in a mouse xenograft model (Fig. 5k, l). Together, these data indicate that loss of FBW7 degrons in the CCDC6-RET fusion elevates its oncogenic phenotype.

### PML-RARA escapes from β-TRCP-mediated degradation

Our systematic bioinformatic analyses of internal degrons relied on previously reported motifs for E3 ligases. However, we and others have validated degrons that may sometimes have unconventional motifs, such as the β-TRCP (F-box/WD repeat-containing protein 1A, FBXW1) degron in Twist (sSspvS)^58^, PER1(tSgcsS)^59^, and CHK1 (tSggcS)^60^. Given drugs that induce protein degradation of PML-RARA lead to high response rates in acute promyelocytic leukemia (APL)^61–65^, we hypothesized that the PML-RARA fusion may escape protein degradation through degron loss, but was missed in our systematic analysis. Interestingly, when using an unconventional β-TRCP degron motif (SSSxxS) reported from a previous study^58^, we found PML-RARA may lead to loss of a degron that is originally found in the PML protein (560-SSSEDS-565) (Supplementary Fig. 5a–c). Among all the genetic fusions observed in TCGA, PML-RARA is the second most frequent fusion event and is preferentially found in LAML^11^. Although not included in TCGA, nearly all APLs contain a PML-RARA fusion (95% of cases), which is caused by the reciprocal translocation t(15;17)(q24;q21)^66^ (Supplementary Fig. 5a). Depending on the exact location of the translocation, PML-RARA fusion yields two major fusions proteins, namely PML-RARa-s and PML-RARa-l (Supplementary Fig. 5b).

Because of the high prevalence and therapeutic relevance of PML-RARA fusions, we next sought to experimentally validate PML as a bona fide substrate of β-TRCP and thereby implicate the predicted degron loss mechanism. Indeed, depleting endogenous *ΒTRC (*also known as *β-TRCP)*, but not other F-box E3 ligase we tested, induced the accumulation of the endogenous PML protein (Supplementary Fig. 5d). In addition, depletion of *β-TRCP* extended the half-life of PML protein (Supplementary Fig. 5e). Consistent with the required phosphorylation of a β-TRCP degron, all four Serine residues were observed to be phosphorylated in a previous unbiased screen^67,68^. Furthermore, depletion of *CSNK2A1* (also known as *CKII*)^69^ also led to the accumulation of PML protein (Supplementary Fig. 5f), indicating that CKII is a potential kinase for PML. Using an in vitro phosphorylation assay, we found that mutation of serine residues within the putative β-TRCP degron (PML-4A) abolished the phosphorylation mediated by CKII kinase (Supplementary Fig. 5g). Moreover, the non-phosphorylated PML mutant (PML-4A) lost the interaction with β-TRCP, thus becoming resistant to β-TRCP-mediated degradation (Supplementary Fig. 5h). Taken together, these results indicate that PML is likely a bona fide substrate of β-TRCP, and loss of a β-TRCP degron likely renders greater stability to PML-RARA fusions.

### Comprehensive analysis of C-terminal degron loss upon oncogenic gene fusion

**Fig. 6:**
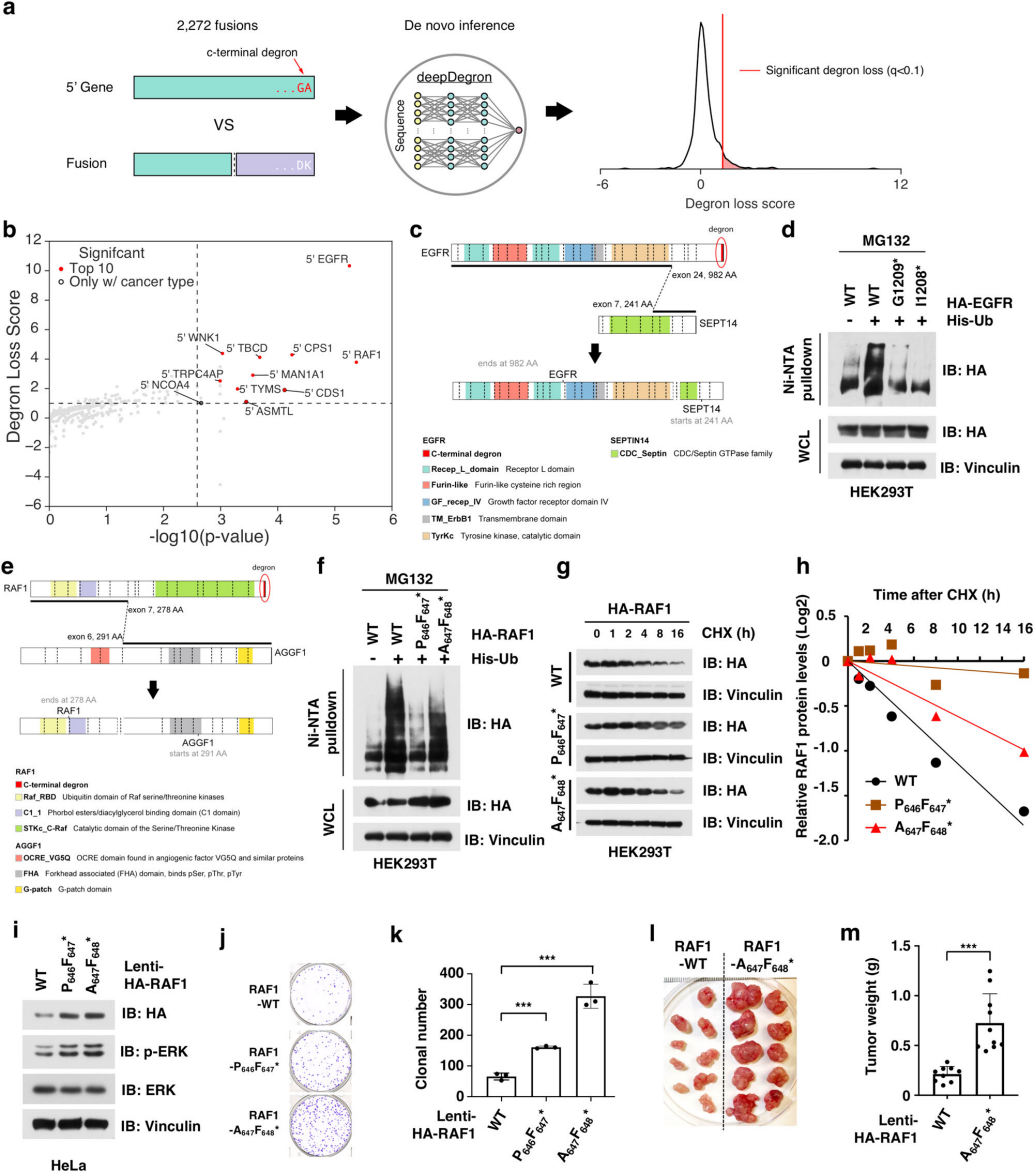
Systematic analysis of C-terminal degron loss. **a** Conceptual diagram showing that fusions resulting in C-terminal degron loss were identified by the deepDegron method. **b** Top fusions enriched for C-terminal degron loss. *X*-axis represents the combined *p* value for both degron loss and cancer type-specificity (Fisher’s method), while the *Y*-axis represents the effect size for degron loss. *P* values were calculated using a one-sided permutation test. See “Methods” for additional details on calculation of *p* values. **c** Diagram of an EGFR-SEPT14 fusion leading to loss of the C-terminal -GA* degron. **d** Removal of C-terminal degron (-GA*) lead to less ubiquitination of EGFR. WT and mutant HA-tagged EGFR proteins were expressed with His-Ub in HEK293T cells and treated with 10 μM MG132 for 12 h. Cells were lysed under denaturing conditions and His-Ub-conjugated proteins were pulled down with Ni-NTA-resin, washed, and immunoblotted for indicated proteins. **e** Diagram of an RAF1-AGGF1 fusion leading to loss of the C-terminal -Vx* degron. **f** Removal of C-terminal degron (-Vx*) lead to less ubiquitination of RAF1. WT and mutant HA-RAF1 were expressed with His-Ub in HEK293T cells and subsequently treated and pulled down as described in **d**. **g**, **h** Removal of C-terminal led to extended protein half-life of RAF1 in cycloheximide (CHX) assay. WT and mutant HA-RAF1 proteins were expressed in HEK293T cells and treated with CHX for indicated time. Cells were harvested, lysed, and immunoblotted for indicated proteins (**g**) and quantification (**h**). **i** Immunoblot analysis for HeLa stable cell lines that express either WT RAF1 or RAF1 mutants without -Vx* degron. **j**, **k** The stable cells expressing the RAF1 mutants without -Vx* degron had greater clonogenicity than WT RAF1-expressing cells in vitro. The stable cell lines as in Fig. 6i were subjected for colony formation assay (**j**) and statistical analysis (**k**). *n* = 3/group. Two independent experiments were performed. **l**, **m** RAF1 mutant-expressing cells developed larger tumors than WT RAF1-expressing cells in vivo. The stable cell lines as in **i** were subjected for mouse xenograft assay (**l**) and statistical analysis (**m**). *n* = 9 mice for the RAF1-WT group and *n* = 10 mice for the RAF1-mutant group. Data are presented as mean ± SD. ***p < 0.01; by two-tailed Student’s *t*-test. Two independent experiments were performed for **d**, **f**, and **g**. The relevant raw data and uncropped blots are provided in Source Data.

The loss of a non-canonical degron in PML-RARA highlights that, even for well-studied E3 ligases like β-TRCP, our current knowledge of degron motifs is largely incomplete. This dearth of knowledge may lead to conclusions that overlook the role of degron loss in fusion events. Thus, we hypothesized that unbiased learning of degron motifs from data would reveal additional cases of degron loss in gene fusions. Although systematic profiling of degrons across the entire proteome has not yet been performed, a previous global protein stability (GPS) assay has systematically measured all C-terminal protein sequences for protein stability, which led to the discovery of several novel degron motifs^31^. We therefore leveraged a machine learning model trained on the GPS assay (deepDegron)^30^ to score whether gene fusions preferentially lead to C-terminal degron loss (Fig. 6a and Supplementary Fig. 6a). We found gene fusions overall were substantially enriched for C-terminal degron loss, with statistical significance further improved by including cancer type information (Supplementary Fig. 6b and Supplementary Data 6). 5′ EGFR and 5′ RAF1 fusions yielded the highest scores for C-terminal degron loss among the 16 statistically significant genes (Fig. 6b). EGFR and RAF1 fusions additionally displayed substantial cancer type-specificity, with 65% of 5′ EGFR fusions occurring in gliomas (Supplementary Fig. 6c) and 69% of 5′ RAF1 fusions occurring in thyroid carcinomas (THCAs) (Supplementary Fig. 6d). Interestingly, C-terminal and internal degrons can be simultaneously lost in a gene fusion, as observed for 5′ NCOA4 fused with 3′ RET (Supplementary Data 1 and Supplementary Fig. 6e).

EGFR-SEPT14 is the most frequent EGFR fusion and occurs mostly in glioblastoma (GBM) and low-grade gliomas (LGG). EGFR-SEPT14 fusions result in loss of a putative C-terminal degron (-GA*, Fig. 6c and Supplementary Fig. 6f), which is evolutionarily conserved among species (Supplementary Fig. 6g). To experimentally validate the key role of the -GA* motif in controlling the protein stability of EGFR protein, we generated two EGFR mutants with either deletion of the last alanine residue (G_1209_*) or glycine–alanine dipeptide (I_1208_*, S6F, Supplementary Fig. 6h). Notably, WT EGFR underwent significant ubiquitination, but both EGFR mutants resulted in a dramatic reduction in ubiquitination (Fig. 6d). This supports our computational finding that a C-terminal degron (-GA*) is lost in *EGFR* genetic fusions, which likely lead to increase stability of the resultant fusion proteins.

Among RAF1 fusions, RAF1-AGGF1 is the most frequent fusion, with 3′ partners TRAK1 and PHC3 being observed less frequently. Our computational analysis predicts a putative C-terminal degron in RAF1 that is evolutionarily conserved among species (-Vx*, x means any amino acid, Fig. 6e and Supplementary Fig. 6i, j). Notably, all RAF1 fusions result in the loss of this putative C-terminal degron. To experimentally validate this finding, we mutated the putative RAF1 degron, by either deletion of the valine residue (P_646_F_647_*) or substitution of the valine to alanine (A_647_F_648_*, Supplementary Fig. 6k). Compared to WT RAF1, both mutants exhibited relatively less ubiquitination (Fig. 6f) and an extended protein half-life (Fig. 6g, h). To further assess whether loss of the C-terminal degron in RAF1 affects tumorigenesis, we generated a HeLa cell line that stably express either WT RAF1 or the degron loss mutants of RAF1 (P_646_F_647_* and A_647_F_648_*, Fig. 6i). Cells expressing the degron loss mutant forms of RAF1 were more clonogenic than those expressing WT-RAF1 in vitro in a colony formation assay (Fig. 6j, k). Furthermore, the RAF1 mutant-expressing cells (A_647_F_648_*) generated larger tumors in a mouse xenograft model than those expressing WT-RAF1 (Fig. 6l, m). These experimental results support our computational prediction that RAF1 loses a C-terminal degron (-Vx*) during fusion events, a process likely rendering greater stability to the fusion protein to facilitate tumorigenesis.

## Discussion

While oncogenic gene fusions in human cancers have been extensively cataloged^11,70^, the molecular mechanisms underlying their oncogenicity is incompletely understood. By analyzing more than 9000 tumors across 33 cancer types, we provide a systematic analysis of genetic fusions that demonstrate the prevalence of degron loss as a mechanism to increase the resultant protein stability. Among the 2406 fusion events that are predicted by machine learning to undergo degron loss, we experimentally validated five highly recurrent oncogenic gene fusions for altered protein stability and oncogenicity, thus more than doubling the number of previously validated cases^16–18^. Prior systematic studies have largely focused on transcriptional over-expression of gene fusions caused by the exchange of promoters or enhancers^11,13^. Our results suggest that degron loss is a complementary and generally applicable mechanism by which genetic fusions increase protein expression levels and thus promote tumorigenesis. We note that degron loss is not necessarily mutually exclusive with other previously proposed mechanisms such as promoter swapping, and therefore might act in concert with them to explain the oncogenicity of a gene fusion. For example, genetic fusions that lead to loss of the C-terminal degrons (such as those for RAF1 or EGFR) might simultaneously promote the kinase activity through a similar mechanism of dimerization or oligomerization^5,8,9,14^.

Despite our study providing the most comprehensive examination of degron loss for genetic fusions to date, many instances of degron loss may still be missed for a couple of reasons. First, our analysis still has limited statistical power in identifying enrichment for degron loss in rare fusion events. For example, a previously validated KEAP1 degron in IKBKB^71^ was lost in HOOK3-IKBKB fusions in breast cancer (Supplementary Fig. 3b), but this fusion event did not surpass our stringent false discovery rate cutoff. Secondly, given the incomplete knowledge of degron motifs, we further prioritized likely true degrons by employing machine learning and ensuring requisite post-translational modifications. However, these stringent criteria may also lead to false negatives in degron motifs, such as the lack of a previously reported phosphorylation event in CCDC6 preventing the accurate prediction of a third FBW7 degron. Further basic science efforts to decipher additional degron motifs coupled with an increased throughput of tumor sequencing will be necessary to provide a complete landscape of degron loss for oncogenic fusions.

Our finding that fusion proteins preferentially escape protein degradation by degron loss suggests that tumors may be particularly sensitive to degradation of oncogenic fusions. Indeed, the standard of care for APL harboring the PML-RARA fusion is either all-*trans*-retinoic acid (ATRA) or arsenic trioxide, both of which lead to the degradation of the PML-RARA fusion protein^61–65^. Given recent advance in the development of compounds that induce targeted protein degradation such as PROTACs (PRoteolysis TArgeting Chimeras)^72^, other fusions besides PML-RARA that undergo degron loss could become efficacious therapeutic targets. Notably, compounds that specifically degrade the BCR-ABL and ALK fusions protein have been developed^73–75^. An additional theoretical benefit of degrading fusion proteins is the possibility to overcome acquired resistance mutations to previously used inhibitors, such as imatinib for BCR-ABL^76,77^ and crizotinib for EML4-ALK fusions^78^. Because not all gene fusions undergo degron loss, our analysis may help prioritize the most promising targets for further PROTAC drug development. However, there are numerous questions that deserve further attention. For example, how can we understand the combinatorial impact of degron loss with other simultaneous mechanisms involved in gene fusions? Are there differences in the functional consequences of pharmacological inhibition versus degradation of stable fusion proteins? Could induced degradation of otherwise stable fusion proteins increase the presentation of neoantigens that yield an immune response against cancer? Future studies of gene fusions that combine mechanistic and bioinformatic insights may reveal the answers to these and more questions.

## Methods

### Human cell lines and culture conditions

Human embryonic kidney 293 (HEK293), HEK293T, HeLa, DU145, and LNCaP cells were purchased from American Type Culture Collection (ATCC). *Spop*^*+/+*^ and *Spop*^*−/−*^ MEFs were kind gifts from Dr. Nicholas Mitsiades (Baylor College of Medicine). The panel of colon cancer cell lines (Lim2405, RKO, DiFi, SW480, Lim1215, LoVo, LS411N, SW1463, SW48, SNU-C2B, HCT8, and SW837) were obtained from Dr. Lin Zhang (University of Pittsburg), and HCT116-*FBW7*-KO, HCT116 WT, and DLD1-*FBW7*-KO, DLD1-WT cell lines were kind gifts from Dr. Bert Vogelstein (John Hopkins University). HEK293, HEK293T, HeLa cells, *Spop*^*+/+*^, and *Spop*^*−/−*^ MEFs were maintained in Dulbecco’s modified Eagle’s medium (DMEM) containing 10% fetal bovine serum (FBS), 100 units of penicillin and 100 µg/ml streptomycin. DU145, LNCaP, HCT116, DLD1, Lim2405, RKO, DiFi, SW480, Lim1215, LoVo, LS411N, SW1463, SW48, SNU-C2B, HCT8, SW837, HCT116-*FBW7*-KO, and DLD1-*FBW7*-KO cells were cultured in RPMI1640 containing 10% FBS, 100 Units of penicillin and 100 µg/ml streptomycin.

### General cloning

Expression vectors HA-ABL1, HA-FBW7, and HA-RAF1 were constructed by cloning the corresponding cDNAs into pcDNA3-HA vector. Flag-SPOP, Flag-SPOP-Y87C, Flag-SPOP-F102C, and Flag-SPOP-W131G were constructed as previous described^17^. Myc-β-TRCP1 was constructed as previous describe^79^. GFP-CCDC6 (571577), GFP-CCDC6/RET (572024), and HA-EGFR (703594) were purchased from MRC PPU (University of Dundee). HA-ABL1-ΔD, Flag-PML-S518A, Flag-PML-4A, Flag-PML-5A, HA-EGFR-G1029*, HA-EGFR-I1028*, HA-RAF1-P646A647*, and HA-RAF1-A647A648* were constructed using the Site-Directed Mutagenesis Kit (Agilent) following the manufacturer’s instructions. GST-PML-WT, GST-PML-4A, and GST-PML-S518A were constructed by cloning the corresponding cDNA into pGEX-GST-4T1 vector. pLenti-HA-CCDC6, pLenti-HA-CCDC6-RET, pLenti-HA-RAF1, pLenti-HA-RAF1-P646A647*, and pLenti-HA-RAF1-A647A648* were constructed by cloning the corresponding cDNAs into pLenti-puro vector. The primers for site mutation are as below: PML-S518A-f: 5′-GCACCTCCAAGGCAGTCGCACCACCCCACCTGG-3′; PML-S518A-r: 5′-CCAGGTGGGGTGGTGCGACTGCCTTGGAGGTGC-3′; PML-4A-f: 5′-CGCGTTGTGGTGATCGCCGCCGCGGAAGACGCAGATGCCGAAAACTCG-3′; PML-4A-r: 5′-CGAGTTTTCGGCATCTGCGTCTTCCGCGGCGGCGATCACCACAACGCG-3′; ABL1-ΔD-f: 5′-GCAAATCCAAGAAGGGGAGCTGTTATCTGGAAG-3′; ABL1-ΔD-r: 5′-CTTCCAGATAACAGCTCCCCTTCTTGGATTTGC-3′; EGFR-G1029-f: 5′-CAGTGAATTTATTGGATGAGCGGCCGCTTACC-3′; EGFR-G1029-r: 5′-GGTAAGCGGCCGCTCATCCAATAAATTCACTG-3′; EGFR-I1028-f: 5′-CAAAGCAGTGAATTTATTTGAGCGGCCGCTTACCC-3′; EGFR-I1028-f: 5′-GGGTAAGCGGCCGCTCAAATAAATTCACTGCTTTG-3′; RAF1-A647A648-f: 5′-CCCCGAGGCTGCCTATGTTCTAGTTGACTTTGCACC-3′; RAF1-A647A648-r: 5′-GGTGCAAAGTCAACTAGAACATAGGCAGCCTCGGGG-3′; RAF1-P646A647-f: 5′-CCCCGAGGCTGCCTTTCTAGTTGACTTTGCACCTG-3′; RAF1-P646A647-r: 5′-CAGGTGCAAAGTCAACTAGAAAGGCAGCCTCGGGG-3′. The shRNA vectors for SPOP were purchased from Sigma (TRCN0000122224, TRCN0000139181, TRCN0000145024).

### Antibodies

The anti-ABL1 (2862, 1:1000), anti-p27 (3686, 1:1000), anti-DEK (13962, 1:1000), anti-SRC3 (2126, 1:1000), anti-CUL3 (2759, 1:1000), anti-GST (2625, 1:2000), anti-β-TRCP (4394, 1:1000), anti-p-ERK(9101, 1:1000), and anti-ERK (4695, 1:1000) antibodies were obtained from Cell Signaling Technology. Anti-ERG (EPR3864, 1:1000) antibody was obtained from Abcam. Anti-SPOP (16750-1-AP, 1:1000) antibody was obtained from Proteintech. Anti-GFP (A-11122, 1:5000) antibody was obtained from Thermo Fisher. Anti-FBW7 (A301-720A, 1:1000) and anti-PML (A301-167A, 1:1000) were obtained from Bethyl Laboratories. Anti-CCDC6 (sc-100309, 1:1000) and anti-α Tubulin (sc-8035, 1:2000) antibodies were obtained from Santa Cruz Biotechnoloy. Mouse monoclonal anti-HA.11 epitope tag (clone 16B12, 901513, 1:1000) was obtained from BioLegend. Anti-Vinculin (V9131, 1:50000), rabbit polyclonal anti-HA (H6908, 1:3000), mouse monoclonal ANTI-FLAG® M2 (F3165, 1:5000), rabbit polyclonal ANTI-FLAG® (F7425, 1:3000), anti-mouse IgG (whole molecule)-peroxidase (A4416, 1:5000), and anti-rabbit IgG (whole molecule)-peroxidase (A4914, 1:5000) were obtained from Sigma-Aldrich. Mouse monoclonal ANTI-FLAG® M2 affinity agarose gel (A2220) and mouse monoclonal anti-HA-agarose (A2095) were obtained from Sigma-Aldrich.

### Annotation of fusion consequence

We annotated the protein sequence consequence of fusions using the software tool AGFusion^80^. To provide consistent annotation, we chose the Matched Annotation from NCBI and EMBL-EBI (MANE select transcripts v0.9) from GENCODE when possible^81^, or otherwise the longest transcript that is consistent with the fusion junction. Transcript annotations were based on Ensembl release 95 using pyensembl (https://github.com/openvax/pyensembl). Of 25,664 fusions reported in TCGA, 24,239 fusions could be annotated. The PFAM database was used to annotate the impact of fusions on protein domains^82^. Code used to analyze fusion genes can be found on github (https://github.com/ctokheim/fusion_pipeline).

### Annotation of cancer driver genes

A consensus among multiple sources was used to annotate previously implicated cancer driver genes, which included OncoKB (https://www.oncokb.org/, downloaded 4/2020)^83^, The Cancer Genome Atlas (TCGA)^84^, and the Cancer Gene Census (CGC, downloaded 4/9/2020)^85^. For CGC, we excluded genes with only support for germline mutations. For OncoKB, we only used genes that were annotated by OncoKB, rather than including additional genes from other sources. To further distinguish oncogenes versus tumor suppressor genes, we annotated based on the evidence from at least one source and no conflicting interpretations. Given that TCGA has cancer type-specific assessments of oncogene and tumor suppressor genes, we chose based on the most frequent annotation across cancer types.

### Enrichment for in-frame fusions

To analyze whether fusions containing driver genes are biased towards in-frame fusions, we analyzed the odds ratio of in-frame vs out-of-frame fusions. The in-frame status of fusions was determined by the annotation software AGFusion (https://github.com/murphycj/AGFusion)^80^. The log odds ratio was calculated separately for oncogene and tumor suppressor gene fusions, relative to putative passenger fusions that do not contain a gene previously implicated in cancer. In cases where a fusion is composed of both an oncogene and a tumor suppressor, the fusion gene was regarded as an oncogene. The standard error for the log odds ratio was calculated using a normal approximation^86^.

### Protein domain analysis

To analyze whether putatively oncogenic fusions preferentially retain protein domains, we compared the odds ratio that a fusion retained at least one protein domain for implicated driver genes (oncogenes or tumor suppressors) to passenger genes. We used domains from PFAM to annotate whether fusion retained or lost protein domains. Protein domains needed to be at least 25 amino acids long. For cases where the fusion junction interrupted a protein domain, we considered a protein domain as retained in the fusion gene if greater than 50% of the protein sequence was included.

### Motif search for internal degrons

We first curated known degron motifs from eukaryotic linear motifs (ELM) database and other literatures^25^ (Supplementary Data 7). Each motif is represented as a regular expression which describes the allowable amino acid residues at each position. Motifs were then searched against the protein translation of GENCODE transcripts using the python “re” package. When multiple transcripts were available for a gene, the MANE select transcript (v0.9) was used. Some degron motifs require not only a particular protein sequence, but also that certain residues have appropriate post-translational modifications (PTM). Towards this end, we collected all available PTMs in the PhosphoSitePlus database^54^ and filtered motif sequence matches for any requisite PTMs (phosphorylation or acetylation). For the non-standard BTRC degron, we used the regular expression “SSSxxS”. The motif search revealed 32,804 hits across 8623 genes involved in TCGA fusions.

### Machine learning prioritization of internal degron motifs

Because motif instances may happen by chance in the proteome, we wanted further prioritize motifs that are a biologically plausible degron. Previously, we developed a model to predict the potential of a motif to be a degron using a Random Forest algorithm. The model was trained on 83 features from the SNVBox database^87,88^ to distinguish previously reported degrons (*n* = 186)^26^ from random other sequences within the same set of proteins (*n* = 186). Features spanned characterization of evolutionary conservation to biophysical features of amino acid residues within a protein. To summarize features across the multiple amino acid residues in a motif, we took the average of each feature. Evaluated using 20-fold cross-validation, performance as measured by the area under the receiver operating characteristic curve (auROC) was 0.8 out of 1.0 (*p* = 2 × 10^−25^, Mann–Whitney *U* test).

### Internal degron motif filtering

Because degron motifs are generally short, motif matches can happen by chance across the proteome. We therefore filtered motifs that had low potential to actually being a degron according to a Random Forest algorithm (score ≤ 0.6 out of 1.0), see section entitled “Machine learning prioritization of internal degron motifs”. This resulted in keeping 2485 high-likelihood degron motifs for downstream analysis.

### C-terminal degron motif

In contrast to internal degron motifs from the ELM database, C-terminal degron motifs were defined based on de novo inference from the Global Protein Stability (GPS) assay^31^ as previously described^30^. Briefly, the c-terminal sequence of every protein in the proteome is ranked by a degron potential score by the deepDegron method. A binomial model is then used to test for motifs that are statistically enriched in high scoring sequences (*q* < 0.05). This revealed 236 C-terminal degron motifs. Note that C-terminal degron motifs may partially overlap so multiple motif matches in a protein sequence are regarded as the same as a single motif match. All C-terminal degron motifs can also be found in Supplementary Data 8. Documentation for deepDegron is available on readthedocs (https://deepdegron.readthedocs.io/) and source code is available on github (https://github.com/ctokheim/deepDegron).

### Statistical test for degron loss in fusion genes

A permutation-based approach was used to determine whether fusions preferentially lead to degron loss. Since a gene may have different fusion partners that all lead to degron loss (e.g. ETV fusions, Fig. 1), we chose to measure enrichment separately for 5′ and 3′ genes. For internal degrons, each gene involved in a fusion received a degron loss score, representing the sum of scores for degrons lost in the fusion. The degron loss score represents both the confidence that the degron exists and the frequency by which it is lost in fusion events. Basically, each predicted degron in a protein sequence receives a score from a Random Forest machine learning model that reflects the confidence in the prediction. The score of that degron is then summed each time a fusion event leads to its loss. Likewise, for c-terminal degron analysis, each 5′ gene involved in a fusion received a delta degron potential score, representing the difference in degron potential scores between the 5′ gene and 3′ gene of a fusion. Only fusions resulting in in-frame fusions were analyzed, as the loss of degron in an out-of-frame fusion would not lead to increased activity of the fusion product. Additionally, as the previously reported validation rate of fusion calls is 63%^11^, we only analyzed genes involved in at least two fusions to mitigate the impact of spurious calls. The sum of degron loss scores for a gene across multiple fusions was then calculated as the test statistic. The observed scores were then compared to 10,000 permutations, where degron loss scores per fusion were randomly shuffled and the gene-based test statistic was recalculated. The *p* value for a gene’s observed test statistic is calculated as the fraction of permutations that have an equal or greater test statistic. Genes were regarded as statistically significant based on the false discovery rate (*q* < 0.1) using the Benjamini–Hochberg method^32^. Given that oncogene fusions display a significant bias towards in-frame mutations, we only considered genes as degron loss candidates if they had at least 50% of fusions as in-frame. To further prioritize oncogene fusions that have degron loss in the oncogene itself rather than the partner gene, we also included analyses of only genes with a retained protein domain and a restricted hypothesis test analyzing only previously implicated oncogenes.

### Statistical test for cancer type-specificity of fusion genes

Similar to the statistical test for degron loss, we also used a permutation test to evaluate whether genes were involved in fusions preferentially found in particular cancer types. To quantify cancer type-specificity, we used entropy,

1 $${h}_{g}=-\kern-0.3pc{\sum}_{c\in {C}_{g}}{p}_{c}{{{\log }}}_{2}{p}_{c}$$

where ${{{h}}}_{{{g}}}$ is the entropy for gene *g*, *c* reflects a particular cancer type, ${{{C}}}_{{{g}}}$ reflects all cancer types with fusions containing gene *g*, and ${{{p}}}_{{{c}}}$ reflects the fraction of fusions for gene *g* found in cancer type *c*. Lower entropy values represent higher cancer type-specificity. We randomly shuffled the labels for cancer types of the fusions 10,000 times, and recomputed ${{{h}}}_{{{g}}}^{{{i}}}$. The corresponding *p* value was calculated as the fraction of permutations *i* that had an entropy equal to or lower than the observed entropy. Genes were regarded as statistically significant based on the false discovery rate (*q* < 0.1) using the Benjamini–Hochberg method^32^.

### Lollipop diagrams

Lollipop diagrams displaying fusion genes were generated using ProteinPaint (https://pecan.stjude.cloud/proteinpaint)^89^. Fusion junctions were submitted according to their genomic coordinates. Protein domains are shown as colored boxes along the protein sequence.

### Immunoblots and immunoprecipitation (IP)

Cells were lysed in EBC buffer (50 mM Tris pH 7.5, 120 mM NaCl, 0.5% NP-40) supplemented with protease inhibitors (Thermo Fisher) and phosphatase inhibitors (phosphatase inhibitor cocktail set I and II, Calbiochem). The lysates were then resolved by sodium dodecyl sulfate polyacrylamide gel electrophoresis (SDS-PAGE) and immunoblotted with indicated antibodies. For IP, 0.5–1 mg lysates were incubated with the appropriate beads for 4 h at 4 °C. Immuno-complexes were washed four times with NETN buffer (20 mM Tris, pH 8.0, 100 mM NaCl, 1 mM EDTA, 0.5% NP-40) before being resolved by SDS-PAGE and immunoblotted for indicated proteins. These primary antibodies were diluted in 5% BSA in TBST and secondary antibodies were diluted in 5% non-fat milk for immunoblotting analysis. The Quantity One software was used for the quantification of protein band intensity, and graphic and statistical analyses were generated using GraphPad 8.

### In vitro kinase assays

PML in vitro kinase assays were performed as previous reported^90^. Briefly, GST-PML-WT, GST-PML-4A, and GST-PML-S518A were expressed in BL21 *E. coli* and purified using Glutathione Sepharose 4B according to the manufacturer’s instructions (Thermo). One microgram of GST-PML-WT, or GST-PML-4A, or GST-PML-S518A protein were incubated with ^32^P-ATP in the absence or presence of CKII kinase in kinase assay buffer (10 mM HEPES, pH 8.0, 10 mM MgCl_2_, 1 mM dithiothreitol, 0.1 mM ATP). The reaction was initiated by the addition of 10× kinase assay buffer in a volume of 30 μL for 45 min at 30 °C followed by the addition of SDS-PAGE sample buffer to stop the reaction before resolved by SDS-PAGE.

### In vivo ubiquitination assays

Denatured in vivo ubiquitination assays were performed as previously described^17^. Briefly, HEK293T cells were transfected with indicated constructs. Fourty-eight hours after transfection, 30 μM MG132 was added to block proteasome degradation for 6 h and then cells were harvested in denatured buffer (6 M guanidine-HCl, pH 8.0, 0.1 M Na_2_HPO_4_/NaH_2_PO_4_, 10 mM imidazole). After sonication, the ubiquitinated proteins were purified by incubation with Ni-NTA matrices for 3 h at room temperature. The pull-down products were washed sequentially twice in buffer A, twice in buffer A/TI mixture (buffer A: buffer TI = 1:3, v/v) and once in buffer TI (25 mM Tris-HCl, pH 6.8, 20 mM imidazole). The poly-ubiquitinated proteins were separated by SDS-PAGE for immunoblot analyses.

### Protein half-life cycloheximide (CHX) chasing assays

To measure the half-life of ABL1 protein, a CHX-based assay was performed following our previously described experimental procedures^90^. Briefly, cells were treated with 200 μg/ml CHX for indicated time before harvest for immunoblot analysis of indicated proteins.

### Colony formation assays

Stable cell lines were seeded into six-well plates in medium (1,000 cells/well) and cultured for 2–3 weeks until colonies are visible. Then, the colonies were washed once with PBS, fixed with fixation buffer (10% acetic acid, 10% methanol) for 20 min, and then stained with staining solution (0.4% crystal violet, 20% ethanol) for 10 min. After staining, the plates were washed with distilled water and air-dried, and then colonies were counted for statistical analysis.

### Mouse xenograft assays

Five- to six-week-old male nude mice were purchase from Taconic (#NCRNU) for xenograft studies. A total of 1 x 10^6^ cells were re-suspended in 100 µl PBS solution and injected subcutaneously into the mice (*n* = 9 or 10 mice for each group) as described previously^79^. At the end of experiment, mice were sacrificed and tumors were dissected for imaging and weighing. All mouse experiments were approved by the Institutional Animal Care and Use Committee (IACUC, RN150D) at Beth Israel Deaconess Medical Center (BIDMC). The Institute is committed to the highest ethical standards of care for animals used for the purpose of continued progress in the field of human cancer research. All mice were housed in a pathogen-free environment at BIDMC animal facility and were handled in strict accordance with the “Guide for the Care and Use of Laboratory Animals” and the applicable institutional regulations.

### Association of CCDC6-RET with leukocyte fraction

To analyze whether CCDC6-RET fusions were associated with leukocyte infiltration, we utilized a previous estimate of immune infiltration for TCGA tumors^91^. A likelihood ratio test was performed after adjusting for tumor purity from ABSOLUTE (downloaded from https://gdc.cancer.gov/about-data/publications/pancanatlas)^92^, tumor mutation burden, and cancer type.

### Association of fusions events with protein abundance (RPPA)

To analyze whether fusion events were associated with an altered proteome, we correlated the mutation status of fusion genes with protein abundance from reverse phase protein array (RPPA) in TCGA^93^. A Wald test was performed after adjustment for cancer type. Only fusions present in at least three tumors were considered.

### Association of fusions events with transcription factor activity

We hypothesized that fusion events may be associated with altered activity of transcription factors. To quantify activity, we leveraged thousands of transcription factor ChIP-seq profiles in Cistrome DB to identify target genes^33^. Computational analysis was then carried out as previously performed^30^. Briefly, we first analyzed fusion events for differentially expressed genes, after adjusting for tumor purity and tumor subtype. RABIT^34^ was then used to infer the transcription factor regulators that explain the differentially expressed genes by using the transcription factor target genes defined by Cistrome DB. Associations were regarded as significant at a family wise error rate of 0.01. Analysis only considered fusions with at least three events in a cancer type and transcription factors not deemed to be an outlier (see below).

### Defining outlier transcription factors

ChIP-seq data defining the target genes of transcription factors can be of inconsistent quality. We reasoned that ChIP-seq datasets that consistently arise as explaining differentially expressed genes for nearly all fusion events may reflect data artifacts. We therefore performed outlier analysis using robust covariance estimation (scikit learn python package)^94^, assuming a gaussian distribution and a significant contamination rate of 0.01 (Supplementary Fig. 3f).

### Reporting summary

Further information on research design is available in the Nature Research Reporting Summary linked to this article.

## Supplementary information

Supplemental Figure S1-S6

Description of additional supplementary files

Dataset 1

Dataset 2

Dataset 3

Dataset 4

Dataset 5

Dataset 6

Dataset 7

Dataset 8

Reporting Summary

## Source data

Source data

## Data Availability

Data are available in the article, Supplementary Information, or Supplementary Data 1–8. The full list of recurrent genetic fusions, full list of genes and oncogenes with internal and C-terminal degron loss, full list of protein abundance of fused genes, full list of downstream transcription factors due to genetic fusions are included in the Supplementary Data. The original gene fusion calls were obtained from Supplementary Data 1 of Gao et al.^11^. The subsequently annotated and processed gene fusion data for downstream statistical analysis is available on GitHub (https://github.com/ctokheim/fusion_pipeline). The output from the analysis can be found in the Supplementary Data. All data used in the analyses described in this study are freely available within the public database, including TCGA (https://www.cancer.gov/tcga), OncoKB (https://www.oncokb.org/), CGC (https://cancer.sanger.ac.uk/census), Uniprot (https://www.uniprot.org/), PFAM (http://pfam.xfam.org/), ELM (http://elm.eu.org/), and PhosphoSitePlus (https://www.phosphosite.org/). Source data are provided with this paper.

## Code availability

Custom code for this manuscript is available on GitHub (https://github.com/ctokheim/fusion_pipeline) and is archived on Zenodo^95^.The README file in the GitHub repository describes how to reproduce the analysis. The code uses python 3 and exact version numbers of dependencies are listed in the environment configuration file. The deepDegron code to analyze c-terminal degrons is also freely available on GitHub (https://github.com/ctokheim/deepDegron).
