# Author Correction: Genetic fusions favor tumorigenesis through degron loss in oncogenes

**DOI:** 10.1038/s41467-025-62490-7

**Published:** 2025-07-31

**Authors:** Jing Liu, Collin Tokheim, Jonathan D. Lee, Wenjian Gan, Brian J. North, X. Shirley Liu, Pier Paolo Pandolfi, Wenyi Wei

**Affiliations:** 1 Department of Pathology, Beth Israel Deaconess Medical Center, Harvard Medical School, Boston, MA 02215 USA; 2 Department of Data Science, Dana-Farber Cancer Institute, Boston, MA 02215 USA; 3 Department of Biostatistics, Harvard T.H. Chan School of Public Health, Boston, MA 02115 USA; 4 Cancer Research Institute, Beth Israel Deaconess Cancer Center, Department of Medicine and Pathology, Beth Israel Deaconess Medical Center, Harvard Medical School, Boston, MA 02215 USA; 5 Paulson School of Engineering and Applied Sciences, Harvard University, Cambridge, MA 02138 USA; 6 Department of Biochemistry and Molecular Biology, Medical University of South Carolina, Charleston, SC 29425 USA; 7 Department of Biomedical Sciences, Creighton University, Omaha, NE 68178 USA; 8 Department of Molecular Biotechnology and Health Sciences, University of Turin, Turin, 10124 Italy; 9 Renown Institute for Cancer, Nevada System of Higher Education, Reno, NV 89502 USA

**Keywords:** Cancer genomics, Ubiquitin ligases, Ubiquitylation, Cancer genetics, Oncogenes

Correction to: *Nature Communications* 10.1038/s41467-021-26871-y, published online 18 November 2021

In the version of this article initially published, there were errors in Fig. 4d and 4h and the associated Source data. In Fig. 4d, the western blot showing Tubulin was incorrect and inadvertently duplicated from Fig. 4h. The correct western blot showing Vinculin for Fig. 4d has been identified and the error corrected in the HTML and PDF versions of the article. The raw data for Fig. 4d and 4h are included as Supplementary information alongside the online version of this amendment.

## Supplementary information

Raw data for Fig. 4d, h

