# Supplementary material for: Genetic fusions favor tumorigenesis through degron loss in oncogenes
Source: Nat Commun. 2021 Nov 18;12:6704. doi: 10.1038/s41467-021-26871-y (PMC8602260; doi:10.1038/s41467-021-26871-y)
Supplement: Supplementary file 1 — Supplemental Figure S1-S6 [file 41467_2021_26871_MOESM1_ESM.pdf]

Supplemental Information

Genetic Fusions Favors Tumorigenesis Through Degron Loss in Oncogenes

Jing Liu, Collin Tokheim, Jonathan D. Lee, Wenjian Gan, Brian J. North,

X Shirley Liu, Pier Paolo Pandolfi, Wenyi Wei

Supplementary Fig. 1. ETVs and ERG fusions escape from COP1- or SPOP-mediated degradation.

Supplementary Fig. 2. Prediction pipeline for degron loss or gain in fusion protein derived from human cancer samples.

Supplementary Fig. 3. Degron loss has cancer type-specificity and downstream functional consequences.

Supplementary Fig. 4. Loss of internal degron lead to stabilization of proteins.

Supplementary Fig. 5. PML-RARA fusion escapes from β-TRCP-mediated degradation in part due to degron loss.

Supplementary Fig. 6. C-degron loss during genetic fusion.

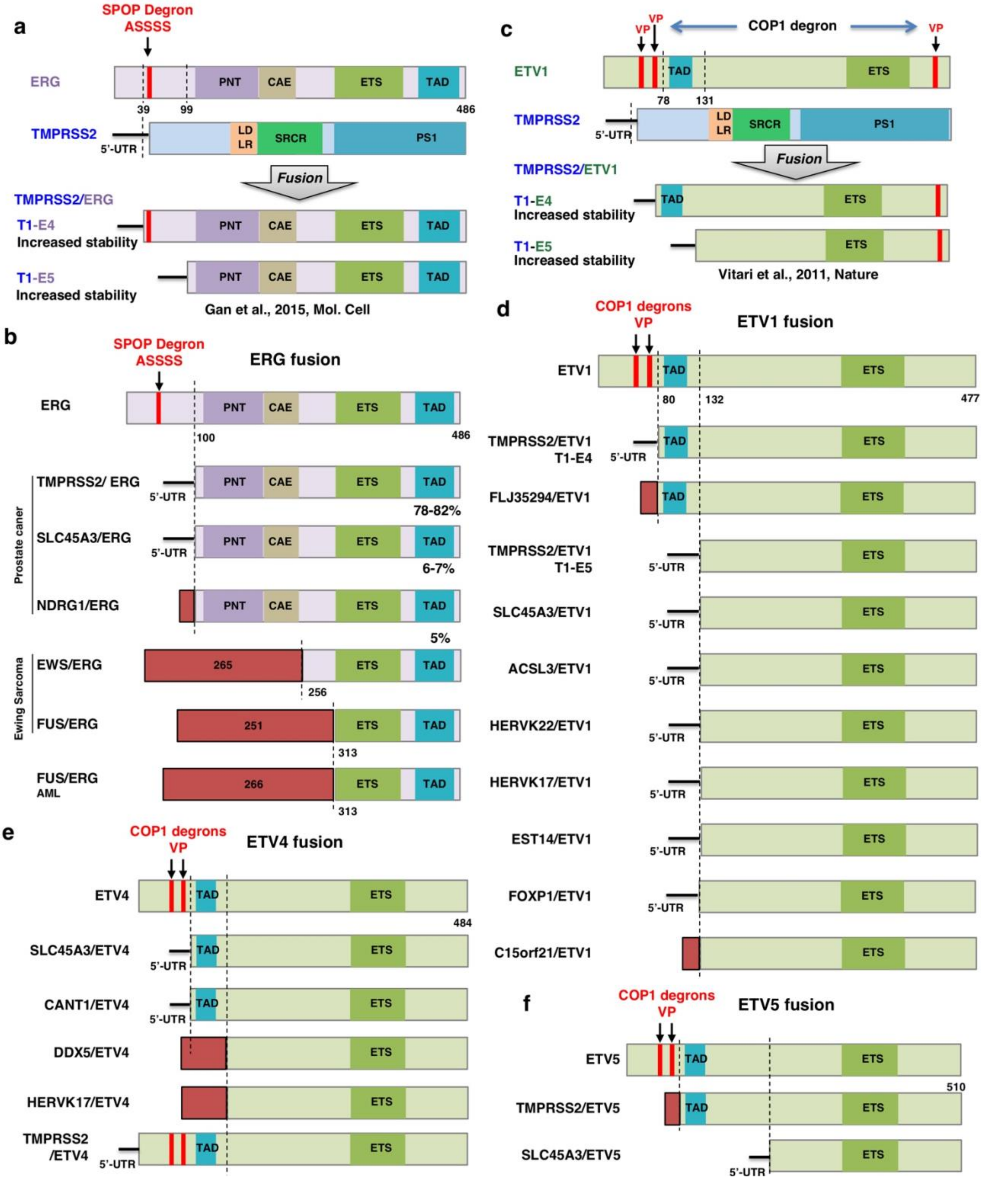

Supplementary Fig. 1. ETVs and ERG fusions escape from COP1- or SPOP-mediated degradation. **(a)** TMPRSS2-ERG fusion leads to the loss of SPOP degron (-ASSSS-) on the N-terminus of ERG protein in T1-E5 isoform. **(b)** A schematic diagram shows the fusion of ERG with several 5' partners, where the fusion proteins escape from SPOP-mediated degradation. The SPOP degron (-ASSSS-) in ERG is lost during gene fusion with TMPRSS2, SLC45A3 or NDRG1 in prostate cancer, as well as

with EWS or FUS in Ewing sarcoma (ES) or AML, respectively. **(c)** TMPRSS2-ETV1 fusion leads to the loss of the two COP1 degrons (-VP-) on the N-terminus of ETV1 protein in both T1-E4 and T1-E5 isoform. **(d)** A schematic diagram shows the fusion of ETV1 with several 5' partners, where the fusion proteins escape from COP-mediated degradation in prostate cancer. The two COP degrons (-VP-) in ETV1 is lost during gene fusion with TMPRSS2, SLC45A3, FLJ35294, ACSL3 and other 5' partners in prostate cancer. **(e-f)** A schematic diagram shows the fusion of *ETV4* genes (E) or *ETV5* genes (F) with other 5' partners in prostate cancer leads to the loss of the two COP1 degrons (-VP-) on the N-terminus of ETV4 or ETV5 proteins.

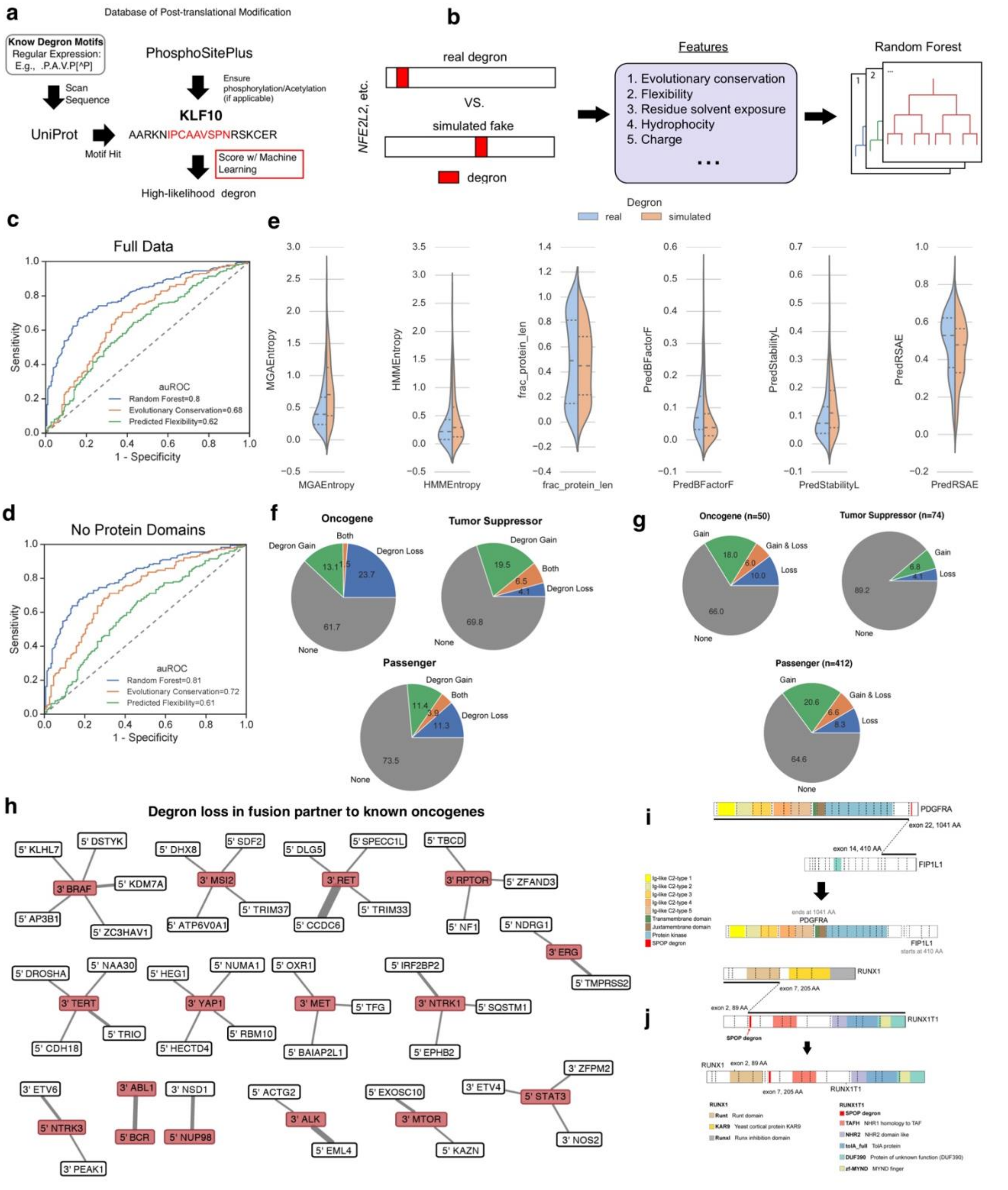

Supplementary Fig. 2. Prediction pipeline for degron loss or gain in fusion protein derived from human cancer samples. (a) A schematic diagram shows the loss or gain of degron during genetic fusion. **(b)** Strategy for high-likelihood degron screen. (**c**) Performance evaluation using the Receiver Operating Characteristic (ROC) curve for the full benchmark. Evolutionary conservation is the negative entropy of a 100-species multiple sequence alignment, predicted flexibility is the predicted B factor for that

location within the protein. (**d**) ROC curve for only examples that are outside of globular protein domains (uniprot). **(e)** Violin plot comparing six informative features used to distinguish true from simulated degrons. Dashed lines indicate the 1st, 2nd and 3rd quartile. **(f-g)** Pie chart indicating the proportion of tumor samples (**f**) or normal samples (**g**) with fusions leading to degron loss or gain. Only recurrent fusions (>5 tumors) are shown. **(h)** Network diagram showing fusion partners to known oncogenes that undergo degron loss. Line width represents the frequency of the event. Red indicates previously annotated oncogenes. Only oncogenes with at least 3 fusion events undergoing degron loss in their partner are shown. (**i**) Diagram of a PDGFRA-FIP1L1 fusion found in a low grade glioma tumor sample. **(j)** Diagram of the RUNX1-RUNX1T1 fusion leading to the addition of a SPOP degron to the tumor suppressor gene RUNX1. The relevant raw data are provided in Source Data.

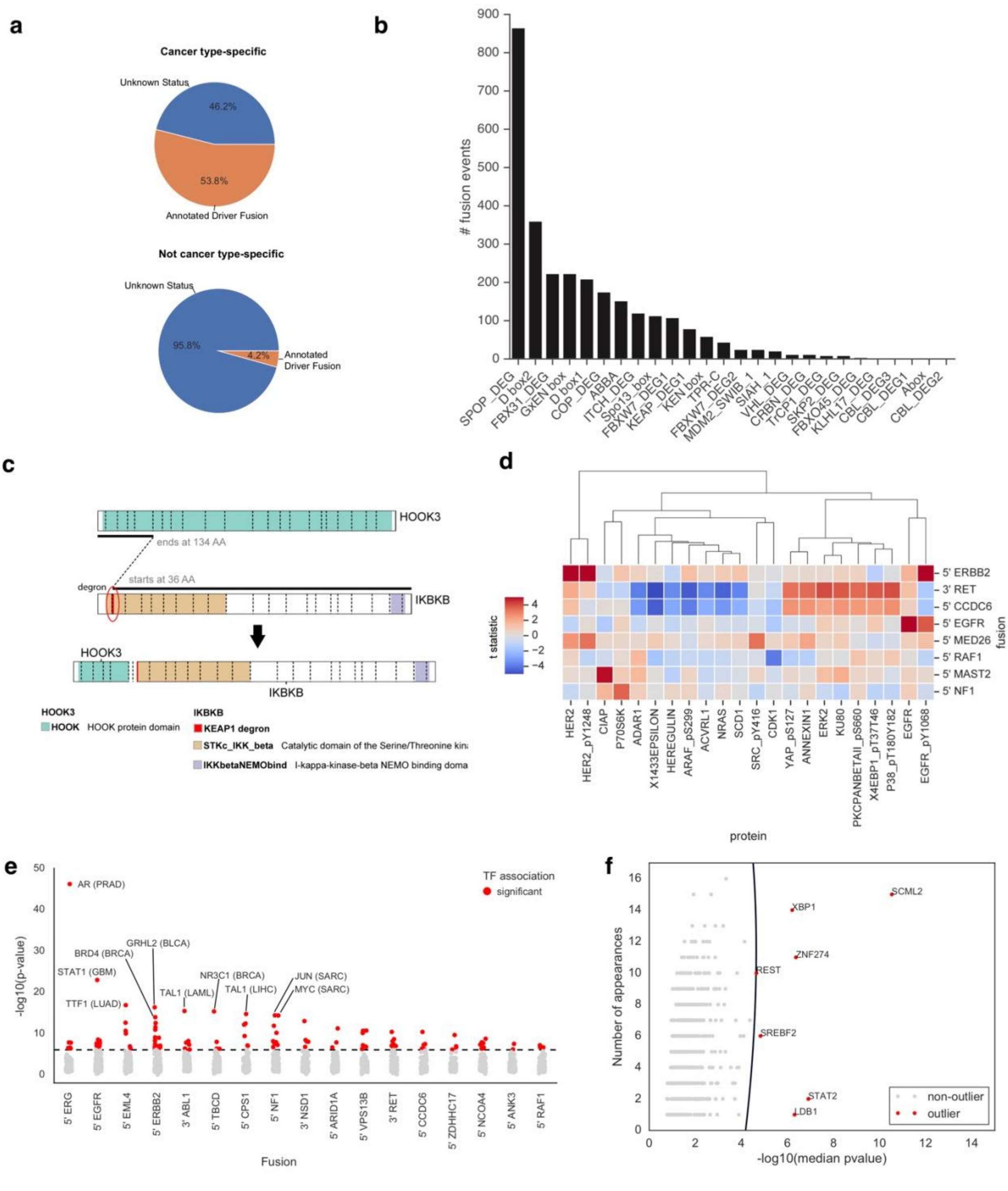

Supplementary Fig. 3. Degron loss has cancer type-specificity and downstream functional consequences. (**a**) Pie chart indicating the percentage of fusion genes identified as cancer type-specific that are previously known to be oncogenic. (**b**) The frequency of fusion events with the loss of specific degron. (**c**) A schematic diagram shows the fusion of *HOOK3* and *IKBKB* genes in breast cancer. **(d)** Heatmap displaying the differential protein abundance of fusion events with enrichment for degron loss. A positive t-statistic indicates an increase in protein abundance (red), while a negative t-statistic indicates a decrease in protein abundance (blue). Only fusions with at least one significant association

are shown (FDR<0.1). (**e**) Association of transcription factor activity with fusion events using the method RABIT. The top 10 most significant associations are labeled, with the cancer type indicated in parentheses. See section “Association of fusions events with transcription factor activity” in Methods for details of p-value calculation. (**f**) Outlier analysis of transcription factors (see section “Defining outlier transcription factors” in Methods). The number of appearances is how many times the transcription factor was identified as a regulator by RABIT, and the p-value also derives from RABIT. The relevant raw data are provided in Source Data.

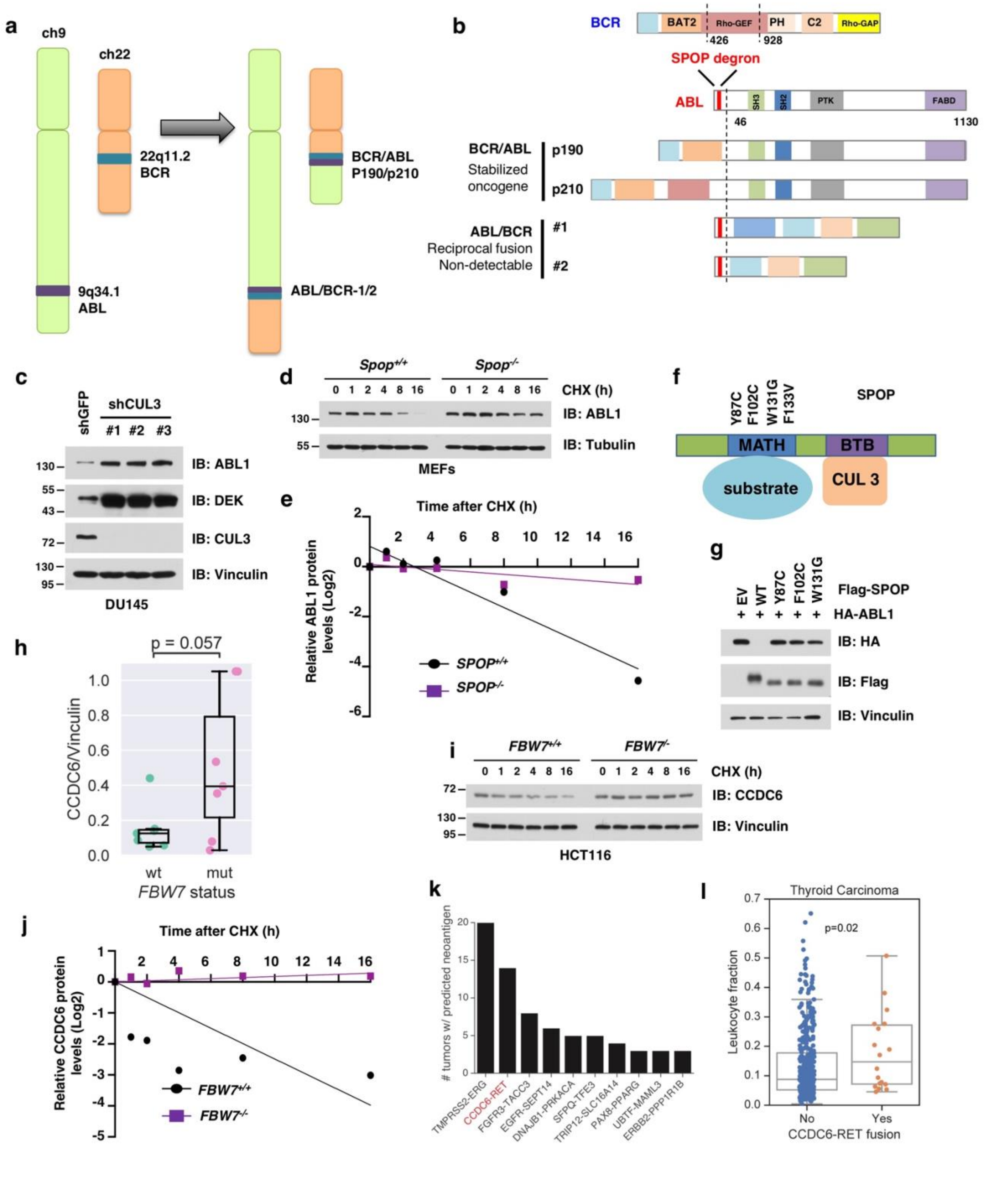

Supplementary Fig. 4. Loss of internal degron lead to stabilization of proteins. (a) A schematic diagram shows the fusion of *BCR* and *ABL* genes in CML due to chromosome arrangement between ch9 and ch22. **(b)** A schematic diagram shows the fusion of *BCR* and *ABL* genes in CML lead to the

generation of two major fusion proteins, p190 and p210. **(c)** Depletion of endogenous *CUL3* leads to accumulation of ABL1. LNCaP cells were infected with shGFP or shCUL3 lenti-virus, and selected with puromycin for 72 hours. Then the cells were harvested for IB of ABL1, with known SPOP substates, ERG as a positive control. This experiment has been repeated twice. **(d-e)** The half-life of ABL protein was extended in *Spop* null MEFs, compared with WT MEFs. Cells were treated with CHX for indicated time, and then harvested for IB (**d**) and quantification (**e**) of ABL proteins. **(f)** A schematic diagram shows the somatic SPOP mutant in its MATH domain, which is responsible for substrate recruiting. **(g)** SPOP mutant in its MATH domain is incapable of degrading ABL1 in cells. HEK293T cells were transfected with HA-ABL1 and indicated Flag-SPOP constructs, and harvested for IB of indicated proteins. **(h)** Quantification of CCDC6 in a panel of CRC cells with WT or mutated *FBW7* genetic background. *FBW7-WT* cells are in green and *FBW7*-mutant cells are in purple. n=7 cell lines for *FBW7-WT* cells group and n=7 cell lines for *FBW7*-mutant cells group. P-value is from a two-sided t test. Boxplot shows the first quartile, median and third quartile, with whiskers extending to +/- 1.5 times the interquartile range. **(i-j)** The half-life of CCDC6 protein was extended in *FBW7* null cells. HCT116 cells with either *FBW7-WT* or *FBW7-KO* genetic background were treated with CHX for indicated time, and then harvested for immunoblot (**i**) and quantification (**j**) of CCDC6 proteins. **(k)** The top 10 most frequent fusion events predicted to generate a neoantigen capable of binding the patient's HLA type. **(l)** Box plot displaying the association of CCDC6-RET fusions and leukocyte fraction in thyroid carcinoma. Boxplot shows the first quartile, median and third quartile, with whiskers extending to +/- 1.5 times the interquartile range. The relevant raw data and uncropped blots are provided in Source Data.

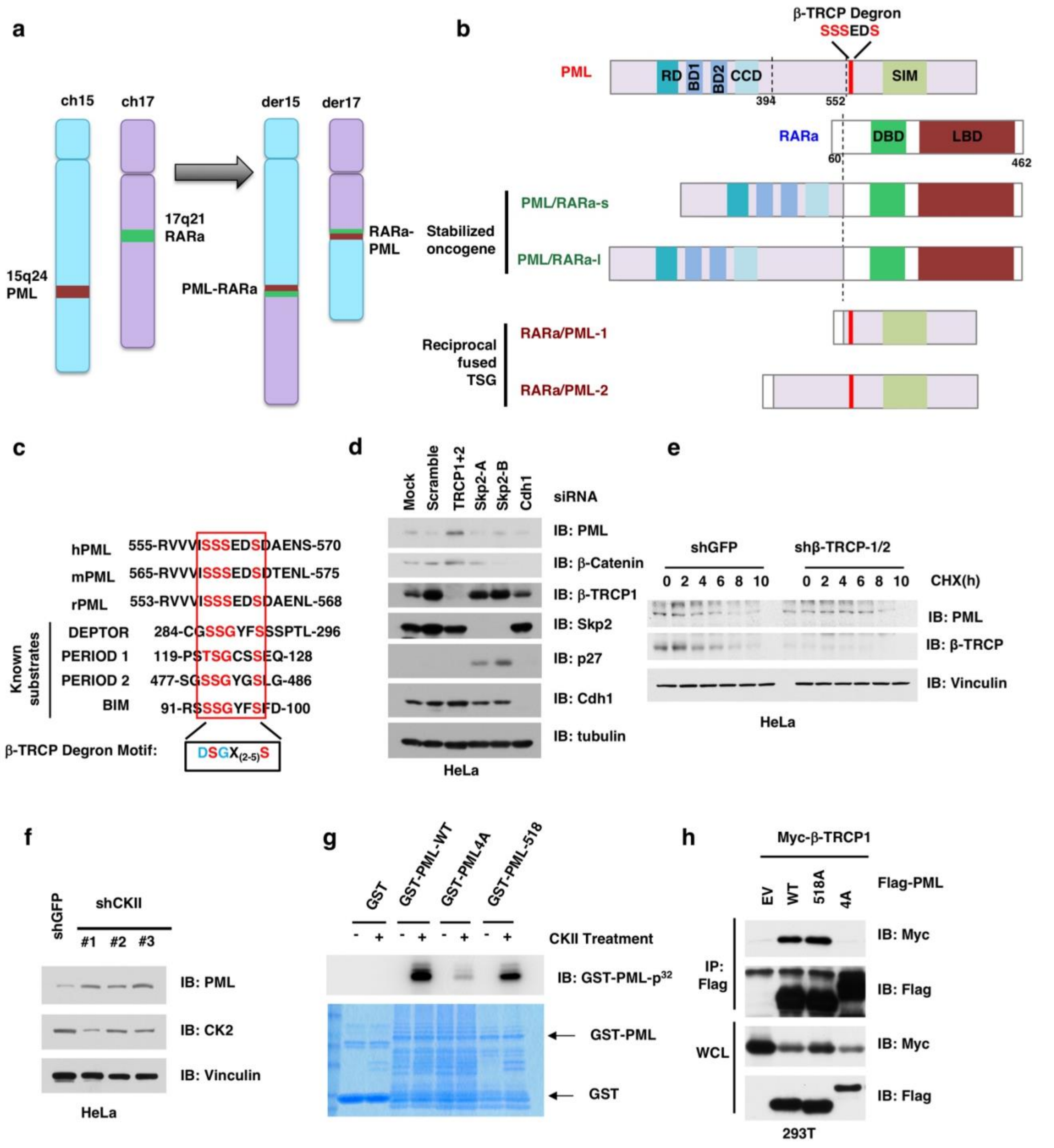

Supplementary Fig. 5. PML-RARA fusion escapes from β-TRCP-mediated degradation in part due to degron loss. (a) A schematic diagram shows the fusion of *PML* and *RARA* genes in chronic myeloid leukemia (CML) due to chromosome arrangement between ch15 and ch17. **(b)** PML-RARA fusion leads to the loss of β-TRCP degron (-SSSEDS-) in PML protein, resulting in stabilization of PML/RARA fusion proteins, PML/RAR-s and PML/RAR-l. In contrast, the reciprocal RARA/PML fusion is destabilized and non-detectable. **(c)** The non-canonic β-TRCP-degron is evolutionarily conserved among species. X: any amino acid. **(d)** Depletion of *β-TRCP* led to accumulation of PML

protein in HeLa cells. **(e)** Depletion of endogenous *β-TRCP* leads to extended protein half-life of PML in HeLa cells. Cells were infected with shGFP or shβ-TRCP lenti-virus, and selected with puromycin for 72 hours. Then the cells were harvested for IB of indicated proteins. **(f)** Depletion of *CKII* kinase led to accumulation of PML protein in H2030 cells. **(g)** CKII kinase phosphorylates the β-TRCP-degron in PML. Bacterial expression GST-PML and mutant proteins were purified and treated with or without CKII kinase in the present of ^{32}P-ATP, and separated by SDS-PAGE. **(h)** Mutation of the β-TRCP-degron in PML abolishes its binding to β-TRCP. IB of the immunoprecipitats (IP) and whole cell lysis (WCL) derived from HEK293T which were transfected with β-TRCP and indicated PML constructs. Two independent experiments were performed for Fig. S5d-h. The relevant raw data and uncropped blots are provided in Source Data.

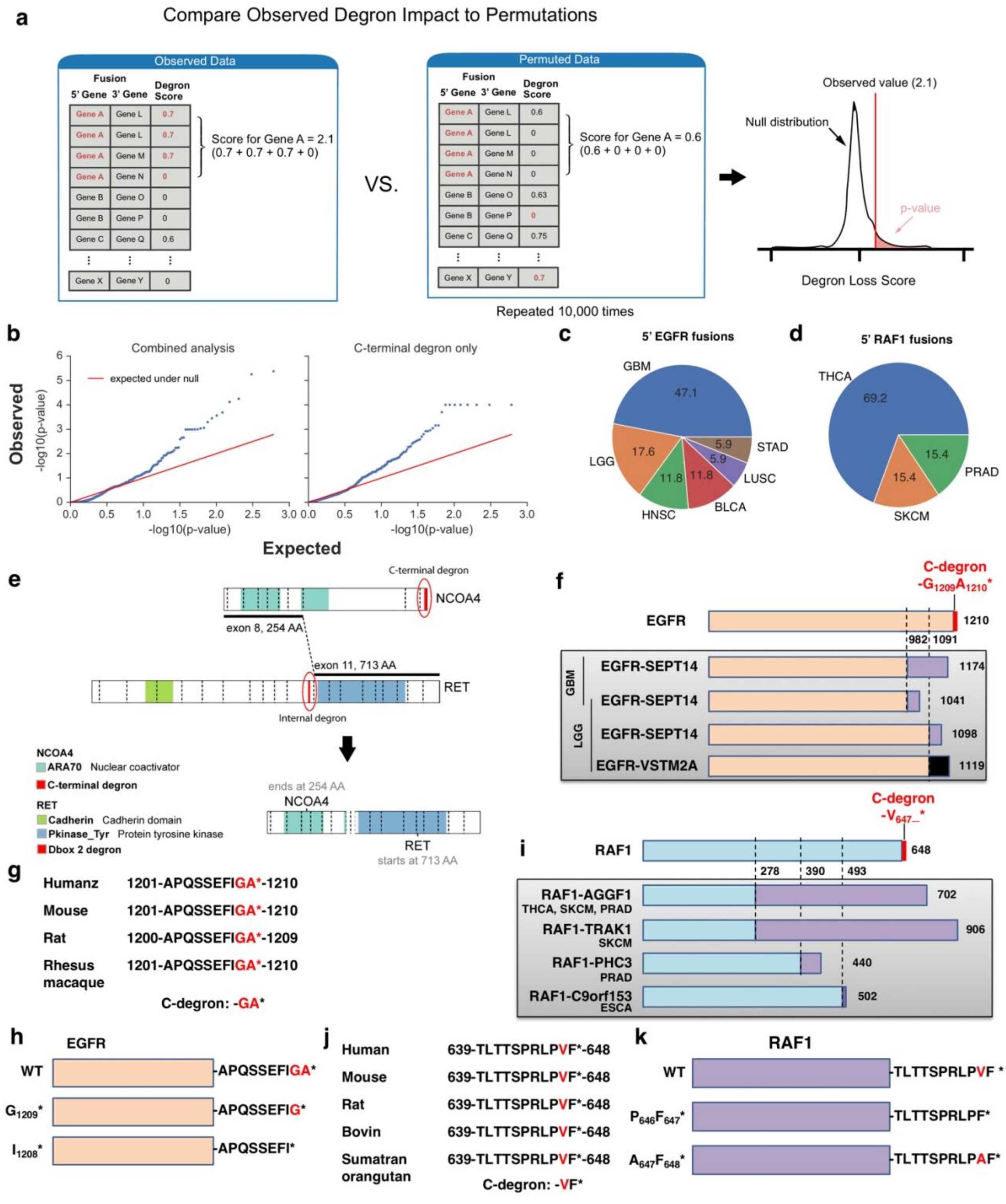

Supplementary Fig. 6. C-degron loss during genetic fusion. (**a**) Diagram illustrating the application of a permutation test to calculate the statistical significance of degron loss. (**b**) Quantile-quantile (QQ) plot illustrating the enrichment for low p-values for the statistical test involving C-terminal degron loss, with (left) and without (right) considering cancer type. P-values were calculated using a one-sided permutation test. See Methods for additional details on calculation of p-values. **(c-d)** Pie chart displaying

the cancer types in which 5’ EGFR (**c**) and 5’ RAF1 (**d**) fusions were found. (**e**) A schematic diagram shows the fusion of *NCOA4* and *RET* genes. **(f)** EGFR fusions led to loss of the putative C-terminal degron (-GA*) in GBM and LGG. **(g)** The C-terminal degron (-GA*) is evolutionarily conserved among species. (**h**) A schematic diagram shows EGFR mutants with either delete of the A_{1210} (G_{1209}*) or $G_{1209}A_{1210}$ residues (I_{1208}*). **(i)** RAF1 fusions led to loss of the putative c-terminal -Vx* degron. **(j)** The C-terminal degron (-Vx*) is evolutionarily conserved among species. (**k)** A schematic diagram shows RAF1 mutants with either deletion of the V_{647} residue ($P_{646}F_{647}$*) or the V647->A mutant ($A_{647}F_{648}$*).
