# Supplementary material for: Genetic fusions favor tumorigenesis through degron loss in oncogenes
Source: Nat Commun. 2021 Nov 18;12:6704. doi: 10.1038/s41467-021-26871-y (PMC8602260; doi:10.1038/s41467-021-26871-y)
Supplement: Supplementary file 2 — Description of additional supplementary files [file 41467_2021_26871_MOESM2_ESM.docx]

**Description of Additional Supplementary Files**

File Name: Supplementary Data 1

Description: List of recurrent genetic fusions in TCGA with potential degron loss.

File Name: Supplementary Data 2

Description: List of genes (A) or oncogenes (B) with internal degron loss during fusions.

File Name: Supplementary Data 3

Description: List of cancer type-specific genes with internal degron loss during fusions.

File Name: Supplementary Data 4

Description: List of genes with C-terminal degron loss during fusions.

File Name: Supplementary Data 5

Description: Protein abundance of fused genes from Reverse Phase Protein Arrays (RPPA) across the TCGA.

File Name: Supplementary Data 6

Description: Increased activity of transcription factors due to genetic fusion events.

File Name: Supplementary Data 7

Description: List of internal degron.

File Name: Supplementary Data 8

Description: List of C-terminal degron.

|  |
| --- |

|  |
| --- |
