# Supplementary material for: Genetic fusions favor tumorigenesis through degron loss in oncogenes
Source: Nat Commun. 2021 Nov 18;12:6704. doi: 10.1038/s41467-021-26871-y (PMC8602260; doi:10.1038/s41467-021-26871-y)
Supplement: Supplementary file 11 — Reporting Summary [file 41467_2021_26871_MOESM11_ESM.pdf]

nature research

Corresponding author(s): Wenyi Wei

Last updated by author(s): Oct 17, 2021

Reporting Summary

Nature Research wishes to improve the reproducibility of the work that we publish. This form provides structure for consistency and transparency in reporting. For further information on Nature Research policies, see Authors & Referees and the Editorial Policy Checklist.

Statistics

For all statistical analyses, confirm that the following items are present in the figure legend, table legend, main text, or Methods section.

n/a	Confirmed	
☐	☒	The exact sample size (n) for each experimental group/condition, given as a discrete number and unit of measurement
☐	☒	A statement on whether measurements were taken from distinct samples or whether the same sample was measured repeatedly
☐	☒	The statistical test(s) used AND whether they are one- or two-sided<br>*Only common tests should be described solely by name; describe more complex techniques in the Methods section.*
☒	☐	A description of all covariates tested
☐	☒	A description of any assumptions or corrections, such as tests of normality and adjustment for multiple comparisons
☐	☒	A full description of the statistical parameters including central tendency (e.g. means) or other basic estimates (e.g. regression coefficient) AND variation (e.g. standard deviation) or associated estimates of uncertainty (e.g. confidence intervals)
☐	☒	For null hypothesis testing, the test statistic (e.g. F, t, r) with confidence intervals, effect sizes, degrees of freedom and P value noted<br>*Give P values as exact values whenever suitable.*
☐	☒	For Bayesian analysis, information on the choice of priors and Markov chain Monte Carlo settings
☐	☒	For hierarchical and complex designs, identification of the appropriate level for tests and full reporting of outcomes
☒	☐	Estimates of effect sizes (e.g. Cohen's d, Pearson's r), indicating how they were calculated

Our web collection on statistics for biologists contains articles on many of the points above.

Software and code

Policy information about availability of computer code

Data collection	No software was used for data collection.
Data analysis	Custom code for this manuscript is available on GitHub (https://github.com/ctokheim/fusion_pipeline) and is archived on Zenodo. The README file in the GitHub repository describes how to reproduce the analysis. The code uses python 3 and exact version numbers of dependencies are listed in the environment configuration file. The deepDegron code to analyze c-terminal degrons is also freely available on GitHub (https://github.com/ctokheim/deepDegron). We used the quantity one software to quantify the protein bands intensity and used the GraphPad 8, to generate the graph figures and statistic analyses. AGFusion software (https://github.com/murphycj/AGFusion) was used for annotating protein sequence consequence of fusions. ProteinPaint (https://pecan.stjude.cloud/proteinpaint) was used for generating Lollipop diagrams.

For manuscripts utilizing custom algorithms or software that are central to the research but not yet described in published literature, software must be made available to editors/reviewers. We strongly encourage code deposition in a community repository (e.g. GitHub). See the Nature Research guidelines for submitting code & software for further information.

Data

Policy information about availability of data

All manuscripts must include a data availability statement. This statement should provide the following information, where applicable:

- Accession codes, unique identifiers, or web links for publicly available datasets
- A list of figures that have associated raw data
- A description of any restrictions on data availability

The PFAM database was used to annotate the impact of fusions on protein domains. A consensus among multiple sources was used to annotate previously implicated cancer driver genes, which included OncoKB, The Cancer Genome Atlas (TCGA), and the Cancer Gene Census (CGC, downloaded 4/9/2020). For CGC, we excluded genes with only support for germline mutations. For OncoKB, we only used genes that were annotated by OncoKB, rather than including additional genes from other sources. Known degron motifs were from eukaryotic linear motifs (ELM) database. For post-translational modification (PTM), data were collected from

the PhosphsitePlus database. The 83 features for Random Forest algorithm model training is from the SNVBox database. The source of all datasets used in the study are detail in the corresponding sections of Methods part. All data used in the analyses described in this study is freely available within the public database, including TCGA (https://www.cancer.gov/tcga), OncoKB (https://www.oncokb.org/), CGC (https://cancer.sanger.ac.uk/census), Uniprot (https://www.uniprot.org/), PFAM (http://pfam.xfam.org/), ELM (http://elm.eu.org/) and PhosphoSitePlus (https://www.phosphosite.org/). Code used to analyze fusion genes can be found on github (https://github.com/ctokheim/fusion_pipeline).Documentation for deepDegron is available on readthedocs (https://deepdegron.readthedocs.io/) and source code is available on github (https://github.com/ctokheim/deepDegron).

Field-specific reporting

Please select the one below that is the best fit for your research. If you are not sure, read the appropriate sections before making your selection.

☒ Life sciences ☐ Behavioural & social sciences ☐ Ecological, evolutionary & environmental sciences

For a reference copy of the document with all sections, see nature.com/documents/nr-reporting-summary-flat.pdf

Life sciences study design

All studies must disclose on these points even when the disclosure is negative.

Sample size	For analyses, 9624 tumor samples of 33 cancer types in TCGA were used, which yield 24,336 fusion genes. Motif search revealed 32,804 hits across 8,623 genes involved in TCGA fusions. Given the large number likely includes matches that happened by chance, we filtered motifs that had low degron potential according to machine learning predictions (<=0.6 out of 1.0). This resulted in keeping 2,485 high-likelihood degron motifs for downstream analysis. The size of animal studies was included in the figure legends. Sample sizes were chosen as large as possible while taking into account the experimental effort required to generate the respective data
Data exclusions	No gene set was excluded during analysis. But for annotating fusions that contain oncogenes and tumor suppressor genes we used CGC and OncoKB, the germline CGC genes were not regarded as cancer driver genes because the scope of this study solely focuses on somatic alterations. Only OncoKB annotations were utilized since other annotations were based on presence in a sequencing gene panel, which does not necessarily indicate those genes are actually cancer driver genes.
Replication	All experimental findings were reliably reproduced. Multiple independent repeats were included for related experiments. Each experiment was performed for at least twice to make sure similar results are reproducible.
Randomization	The training dataset for machine learning does not constitute experimental samples, but rather is based on all known literature annotations compared to completely randomized other positions in the same protein sequence. TCGA has 33 cancer types across 9624 tumor samples, and these samples are dictated by the sample collection of the TCGA consortium (Gao et al., 2018). For the Machine learning, model was trained on 83 features from the SNVBox databaseto distinguish previously reported degrons (n=186) from random other sequences within the same set of proteins (n=186). For statistical test for cancer type-specificity of fusion genes, We randomly shuffled the labels for cancer types of the fusions 10,000 times for analyses. For all in vivo experiments, animals were randomly assigned into different groups for tumor inoculation.
Blinding	For cell-based experiments Western blotting, cell types were known when preparing the samples or starting to treat cells at the beginning of experiments. No blinding was carried out for these in vitro assays. For in vivo animal work, the investigators were not blinded to group allocation during data collection and/or analysis at the end of each experiment.

Reporting for specific materials, systems and methods

We require information from authors about some types of materials, experimental systems and methods used in many studies. Here, indicate whether each material, system or method listed is relevant to your study. If you are not sure if a list item applies to your research, read the appropriate section before selecting a response.

Materials & experimental systems

n/a	Involved in the study
☐	☒ Antibodies
☐	☒ Eukaryotic cell lines
☒	☐ Palaeontology
☐	☒ Animals and other organisms
☒	☐ Human research participants
☒	☐ Clinical data

Methods

n/a	Involved in the study
☒	☐ ChIP-seq
☒	☐ Flow cytometry
☒	☐ MRI-based neuroimaging

Antibodies

Antibodies used	Antibodies were used at a 1:1000 dilution in TBST buffer with 5% non-fat milk for western blot unless specifically indicated below.

The anti-ABL1 (2862), anti-p27 (3686), anti-DEK (13962), anti-SRC3 (2126), anti-CUL3 (2759), anti-GST (2625, 1:2000), anti-β-TRCP (4394), p-ERK(9101) and ERK (4695) antibodies were obtained from Cell Signaling Technology. Anti-ERG (EPR3864) antibody was obtained from Abcam. Anti-SPOP (16750-1-AP) antibody was obtained from Proteintech. Anti-GFP (A-11122, 1:5000) antibody was obtained from Thermofisher. Anti-FBW7 (A301-720A) and anti-PML (A301-167A) were obtained from Bethyl Laboratories. Anti-CCDC6 (sc-100309) and anti-α Tubulin (sc-8035, 1:2000) antibodies were obtained from Santa Cruz Biotechnoloy. Mouse monoclonal Anti-HA.11 epitope tag (clone 16B12, 901513) was obtained from BioLegend. Anti-Vinculin (V9131, 1:50000), rabbit polyclonal anti-HA (H6908, 1:3000), Mouse monoclonal ANTI-FLAG® M2 (F3165, 1:5000), Rabbit polyclonal ANTI-FLAG® (F7425, 1:3000), anti-mouse IgG (whole molecule)-peroxidase (A4416, 1:5000) and anti-rabbit IgG (whole molecule)-peroxidase (A4914, 1:5000) were obtained from Sigma-Aldrich. Mouse monoclonal ANTI-FLAG® M2 affinity agarose gel (A2220) and Mouse monoclonal anti-HA-agarose (A2095) were obtained from Sigma-Aldrich.

Validation

All antibodies used in our study have been validated and detailed information could be found on the website from manufactures as listed below. Some of them have also been validated by our experiments as shown in this manuscript using either over-express, knockout or knockdown strategies.

anti-ABL1 (2862), https://www.cellsignal.com/products/primary-antibodies/c-abl-antibody/2862

anti-p27 (3686), https://www.cellsignal.com/products/primary-antibodies/p27-kip1-d69c12-xp-rabbit-mab/3686

anti-DEK (13962), https://www.cellsignal.com/products/primary-antibodies/dek-e1l3v-rabbit-mab/13962

anti-SRC3 (2126), https://www.cellsignal.com/products/primary-antibodies/src-3-5e11-rabbit-mab/2126

anti-CUL3 (2759), https://www.cellsignal.com/products/primary-antibodies/cul3-antibody/2759

anti-GST (2625), https://www.cellsignal.com/products/primary-antibodies/gst-91g1-rabbit-mab/2625

anti-β-TRCP (4394), https://www.cellsignal.com/products/primary-antibodies/b-trcp-d13f10-rabbit-mab/4394

anti-p-ERK (9101), https://www.cellsignal.com/products/primary-antibodies/phospho-p44-42-mapk-erk1-2-thr202-tyr204-antibody/9101

anti-ERK (4695), https://www.cellsignal.com/products/primary-antibodies/p44-42-mapk-erk1-2-137f5-rabbit-mab/4695

anti-ERG (EPR3864), https://www.abcam.com/erg-antibody-epr3864-ab92513.html

anti-SPOP (16750-1-AP), https://www.ptgcn.com/products/SPOP-Antibody-16750-1-AP.htm

anti-GFP (8371-2), https://www.thermofisher.com/antibody/product/GFP-Antibody-Polyclonal/A-11122

anti-FBW7 (A301-720A), https://www.bethyl.com/product/A301-720A/FBW7+Antibody

anti-PML (A301-167A), https://www.bethyl.com/product/A301-167A/PML+Antibody

anti-CCDC6 (sc-100309), https://www.scbt.com/p/ccdc6-antibody-q-23

anti-α Tubulin (sc-8035), https://www.scbt.com/p/alpha-tubulin-antibody-tu-02

Mouse monoclonal Anti-HA.11 epitope tag (901513), https://www.biolegend.com/en-us/products/anti-ha-11-epitope-tag-antibody-11071

anti-Vinculin (V9131), https://www.sigmaaldrich.com/catalog/product/sigma/v9131

rabbit polyclonal anti-HA (H6908), https://www.sigmaaldrich.com/catalog/product/sigma/h6908

mouse monoclonal ANTI-FLAG® M2 (F3165), https://www.sigmaaldrich.com/catalog/product/sigma/f3165

rabbit polyclonal ANTI-FLAG® (F7425), https://www.sigmaaldrich.com/catalog/product/sigma/f7425

Flag agarose beads (A-2220), https://www.sigmaaldrich.com/catalog/product/sigma/a2220?lang=en&region=US

HA agarose beads (A-2095), https://www.sigmaaldrich.com/catalog/product/sigma/a2095?lang=en&region=US

peroxidase-conjugated anti-mouse secondary antibody (A-4416), https://www.sigmaaldrich.com/catalog/product/sigma/a4416?lang=en&region=US

peroxidase-conjugated anti-rabbit secondary antibody (A-4914), https://www.sigmaaldrich.com/catalog/product/sigma/a4914?lang=en&region=US

Eukaryotic cell lines

Policy information about cell lines

Cell line source(s)	HEK293, HEK293T, HeLa, DU145 and LNCaP cells were purchased from American Type Culture Collection (ATCC). Spop+/+ and Spop-/- MEFs were kind gifts from Dr. Nicholas Mitsiades (Baylor College of Medicine). The panel of colon cancer cell lines (Lim2405, RKO, DiFi, SW480, Lim1215, LoVo, LS411N, SW1463, SW48, SNU-C2B, HCT8, SW837) were obtained from Dr. Lin Zhang (University of Pittsburg) and HCT116-FBW7-KO, HCT116-WT, and DLD1-FBW7-KO, DLD1-WT cell lines were kind gifts from Dr. Bert Vogelstein (John Hopkins University).
Authentication	All cell lines used in this study were authenticated by STR profile report.
Mycoplasma contamination	All cell lines were routinely tested for mycoplasma contamination and cells used in this study were mycoplasma free.
Commonly misidentified lines (See ICLAC register)	N/A

Animals and other organisms

Policy information about studies involving animals; ARRIVE guidelines recommended for reporting animal research

Laboratory animals	The male nude mice at 4-5 week old were purchased from Taconic mouse facility. For Tumor xenograft models, tumor cells were injected subcutaneously into both flanks of 5-6 week old male nude mice. All animal experiments were approved by All experimental procedures were approved by the Institutional Animal Care & Use Committee (IACUC, RN150D) at Beth Israel Deaconess Medical Center with protocol #043-2015. The research projects that are approved by the IACUC are operated according the applicable Institutional regulations. All these mice were randomly allocated into experimental groups.
Wild animals	No wild animals involved in this study.
Field-collected samples	This study didn't involve samples collected from field.
Ethics oversight	All animal experiments were approved by All experimental procedures were approved by the Institutional Animal Care & Use Committee (IACUC, RN150D) at Beth Israel Deaconess Medical Center with protocol #043-2015. The research projects that are approved by the IACUC are operated according the applicable Institutional regulations.

Note that full information on the approval of the study protocol must also be provided in the manuscript.
