# Supplementary figures and images for: Author Correction: Genetic fusions favor tumorigenesis through degron loss in oncogenes
Source: Nat Commun. 2025 Jul 31;16:7014. doi: 10.1038/s41467-025-62490-7 (PMC12313855; doi:10.1038/s41467-025-62490-7)

Raw data for Fig. 4d

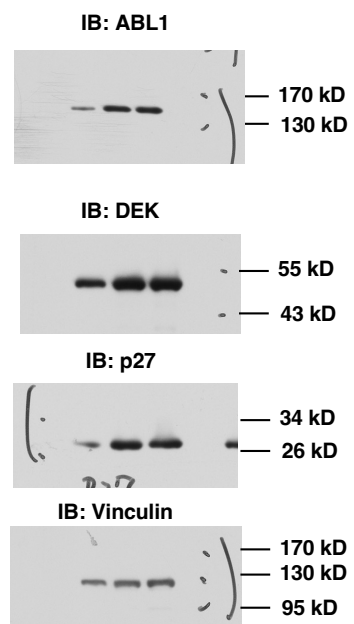

Raw data for Fig. 4h

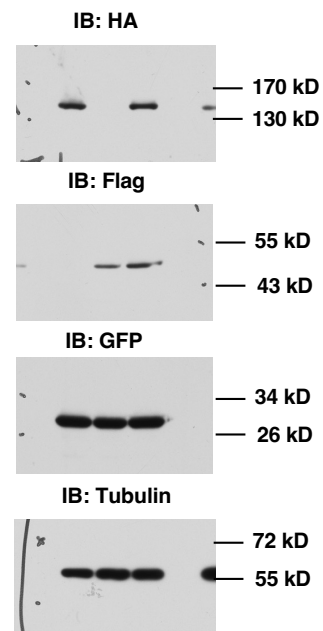

Supplement: Supplementary file 1 — Raw data for Fig. 4d, h [file 41467_2025_62490_MOESM1_ESM.pdf]
